# POSTER PRESENTATIONS

**DOI:** 10.1038/sj.bjc.6601057

**Published:** 2003-06-27


**British Journal of Cancer (2003) 88**(Suppl 1), S55 – S72
© 2003 Cancer Research UK All rights reserved 0007 – 0920/03 $25.00
**www.bjcancer.com**

### POSTER PRESENTATIONS

### P119

5-FLUOROURACIL TOXICITY - AN IMPROVED DETECTION METHOD FOR THE INTRON 14 G TO A MUTATION ASSOCIATED WITH DIHYDROPYRIMIDINE DEHYDROGENASE DEFICIENCY

Susan D. Richman*, Julian W. Adlard. Academic Department of Pathology, Algernon Firth Building, University of Leeds, LS2 9JT.

Dihydropyrimidine dehydrogenase (DPD) is the rate-limiting enzyme in the catabolism of 5-fluorouracil (5FU). DPD deficiency, seen in up to 3% of the population, leads to severe 5FU toxicity and is most often due to a G to A point mutation within the splice-site of intron 14. A published method of identifying this mutation has used PCR followed by digestion with the MaeII restriction enzyme (1). However, this method lacks an internal control for the digestion process. Incomplete digestion of wild type DNA can produce similar results to DNA heterozygous for the mutation. Sequencing of the PCR product may be necessary to confirm the mutation. Also, the MaeII restriction endonuclease is expensive for use in large population or trial-based studies.

We have developed a novel detection method, involving PCR of a 231 base pair product spanning the mutation site, followed by a restriction digestion with Msl1. The method was validated on DNA from known heterozygotes for the mutation (supplied by Dr. Howard McLeod, St. Louis, USA) and wild-type controls. This technique avoids false positive results as there is a splice site present in both the wild-type and mutant DNA which acts as an internal control for complete digestion. A second spice site occurs only when the G-A mutation is present, hence allowing detection of the mutant allele. Heterozygotes and homozygotes for the mutation can be distinguished. The small size of the PCR product makes this method suitable for analysis of DNA from a variety of sources including formalin-fixed paraffin-embedded specimens. The Msl1 restriction endonuclease is approximately 1/20$^{th}$ of the cost per unit compared with MaeII.

Our technique represents a more cost-effective method of screening for the intron 14 DPD mutation associated with 5FU toxicity.

Reference: 1) Wei, X et al. 1996. J Clin Invest. 98:610.

### P120

**WITHDRAWN**

### P121

PHASE 1 STUDY OF MITOMYCIN-C (MMC) & CONTINUOUS INFUSION (CI) 5-FU CHEMORADIATION IN LOCALLY ADVANCED RECTAL CANCER (LARC).

AH Sadozye[1], LM Samuel[2], L McMahon[1] and *AC McDonald[1] for the Colorectal Clinical Oncology Group. [1] Beatson Oncology Centre, Glasgow G11 6NT UK and [2] Aberdeen Royal Hospitals.

**Introduction and aims:** CI 5-FU based chemoradiation (CRT) facilitates tumour downsizing in patients (pts) with LARC. Although 5-FU is often delivered at 225mg/m$^2$/day, 7 days / week, optimal dose/scheduling remains unclear. The addition of MMC to CI 5-FU improves response rates in metastatic disease and MMC is a key component in anal cancer CRT. This phase 1 trial aims to determine the maximum tolerated dose of CI 5-FU / MMC combination CRT in pts with LARC. **Methods:** Pts were accrued sequentially to one of 4 cohorts (3 pts / cohort) and received escalating doses of MMC (IV bolus day 1) and CI 5-FU (7days/week via PICC line). Cohort 1 MMC 8mg/m$^2$ & 5-FU 200mg/m$^2$/day; Cohort 2 MMC 8mg/m$^2$ & 5-FU 225mg/m$^2$/day; Cohort 3 MMC 10mg/m$^2$ & 5-FU 225mg/m$^2$/day; Cohort 4 MMC 10mg/m$^2$ & 5-FU 250mg/m$^2$/day. Pelvic radiotherapy (RT, 45Gy / 25#) was delivered to a CT-planned volume. **Results**: Of 13 pts (med age = 55yrs, 8 males) accrued, 12 have completed treatment. Two pts within cohort 4 had dose limiting toxicity (gd 3 PR bleeding=1pt, gd 3 diarrhoea=1pt). Significant (>gd 1) haematological toxicity was not seen. Eight pts have undergone resection (ypT$_{1-2}$ ypN$_0$=2pts, ypT$_{3-4}$ or ypN+=4pts, pCR=1pt, histology awaited 1pt). All 8 pts had tumour-free circumferential resection margins. Four pts were not resected due to the development of interval metastatic disease.

**Conclusion:** As part of CRT for LARC, MMC 10mg/m$^2$ is deliverable with standard CI 5-FU doses, with acceptable toxicity. Accrual continues at MMC 10mg/m$^2$ + 5-FU 250mg/m$^2$/day.

| **Level** | **n** | **MMC dose** | **5-FU dose** |
|---|---|---|---|
| 1 | 3 | 8mg/m2 | 200mg/m2/day |
| 2 | 3 | 8mg/m2 | 225mg/m2/day |
| 3 | 3 | 10mg/m2 | 225mg/m2/day |
| 4 | 3 | 10mg/m2 | 250mg/m2/day |

### P122

POTENTIATION OF RALTITREXED (RTX) IN *VITRO* BY THYMIDINE PHOSPHORYLASE (TP) AND TP DISTRIBUTION IN *VIVO*

Claire L. Graham*, H. D. Thomas, R. Melton#, G.A.Taylor, D.R Newell, A.V. Boddy. Northern Institute for Cancer Research, University of Newcastle, NE2 4HH. #Enact Pharma Plc, Salisbury.

RTX is a classical thymidylate synthase (TS) inhibitor used to treat colorectal cancer. Inhibition of TS by RTX leads to a dose dependent depletion of dTTP, which is required for DNA synthesis and repair. Tumours may circumvent TS inhibition by salvage of endogenous thymidine (TdR). TP can convert endogenous TdR to thymine and deoxyribose-1-phosphate, thus reducing available TdR for salvage.

In *vitro* 3μM TdR is sufficient to reverse RTX growth inhibition in colorectal carcinoma cell lines. In the presence of TdR, RTX growth inhibition can be potentiated by the addition of 0.005 – 0.025 units/ml of TP.

Mice bearing LoVo and HT29 xenografts were given 1Unit of TP i.v. TP and TdR were measured in the tumour (T) and plasma (P) 24, 48 and 72h after administration of TP. TP was measured as thymine formation nmoles/min/μg protein. TdR was measured as μM in plasma and nmoles/mg protein. Plasma TP was undetectable at each of the time points.

| Hours post TP | | LoVo | | | | HT29 | | | |
|---|---|---|---|---|---|---|---|---|---|
| | | 0 | 24 | 48 | 72 | 0 | 24 | 48 | 72 |
| T | TdR | 0.06 | 0.17 | 0.09 | 0.12 | 0.20 | 0.52 | 0.33 | 0.21 |
| | TP | 0.98 | 0.64 | 0.42 | 0.51 | 0.19 | 0.14 | 0.16 | 0.10 |
| P | TdR | 0.38 | 0.49 | 0.56 | 0.42 | 0.75 | 0.70 | 0.63 | 0.51 |

As the native protein TP does not accumulate in the tumour, in contrast to the results seen when TP is conjugated to an anti-CEA antibody.

### P123

ROLE OF CYCLOOXYGENASE-2 IN LIVER METASTASES ARISING FROM COLORECTAL CARCINOMA

Milind Rao*, Wenxuan Yang, Alexander M. Seifalian, Marc C. Winslet. *University Department of Surgery, Royal Free and University College Medical School, University College London and The Royal Free Hospital, London, UK*

**Aim:** Cyclooxygenase-2 (COX-2) is involved in the development of colorectal carcinoma but its role in liver metastases is not established. We aimed to study the COX-2 expression and the effect of Rofecoxib, a selective COX-2 inhibitor in a rat model of liver metastases. **Methods:** 6 BD1X rats (Control) were inoculated with DHD/K12 colorectal cancer cell line (5 x $10^6$) through intra-portal injection and metastases were examined three weeks after inoculation. In another group, 6 BD1X rats (Rofecoxib) were fed with Rofecoxib (10mg/kg body weight /day) for 3 weeks following inoculation. COX-2 expression in liver tissue and metastatic tumour was detected using conventional and fluorescent immunohistochemistry observed under confocal microscopy. **Results:** Liver metastases developed in all animals three weeks after inoculation. No metastases were seen anywhere outside the liver. There were no differences in the number of metastatic nodules between control and Rofecoxib groups (230 vs. 249). COX-2 was expressed in both the metastatic tumour cells and the hepatocytes (around the central vein and the vicinity of the tumour). Fluorescent staining further supported these findings. Notably, the intensity of staining of tumour tissue was higher than that in the surrounding hepatocytes. Also, COX-2 positive staining was seen mainly in the cytoplasm of metastatic tumour cells while it only presented in the nuclei of the hepatocytes. **Conclusion:** COX-2 is expressed in liver metastases and the surrounding liver tissue. Rofecoxib did not reduce number of liver metastases.

### P124

A RANDOMISED TRIAL OF IRINOTECAN UNTIL DISEASE PROGRESSION (PD) VERSUS 8 CYCLES ONLY IN FLUOROPYRIMIDINE-RESISTANT ADVANCED COLORECTAL CANCER (CRC)

Kumar R. Lal*, Paul J Ross, Andrew R Norman, Alison Massey, Mark Hill, Gary Middleton, Claire Topham,David Cunningham.
Royal Marsden Hospital, Downs Road, Sutton, SM2 5PT.

Irinotecan continued until PD is standard of care for patients with fluoropyrimidine-resistant CRC. We evaluated the optimal duration of irinotecan therapy in a randomised trial comparing stopping treatment after 8 cycles to continuing until PD. 333 patients (pts) with locally advanced or metastatic CRC that had PD on or within 24 weeks of completion of fluoropyrimidine therapy received irinotecan 350mg/m$^2$ 3-weekly. After 8 cycles 230 pts had PD, 30 stopped due to toxicity, 2 refused randomisation but continued irinotecan and 2 were withdrawn. 55 pts with stable or responding disease were randomised, 25 pts, including 6 responders, to continue irinotecan and 30 pts, including 8 responders, to discontinue irinotecan. The mean age of randomised pts was 62.4 years (range 42-78) and demographics were balanced between the 2 cohorts. A total of 277 additional cycles were delivered to the continue irinotecan group, with a median of 12 cycles in total (range 9-20). There were no additional responses after randomisation. Grade 3/4 diarrhoea occurred in 8%; there was no grade 3/4 febrile neutropaenia. 16 patients randomised to stop irinotecan had further chemotherapy at PD, 8 with irinotecan. PFS at 6 months was 36.4% (95% CU 17.4-55.4%) in the continue irinotecan arm and 25% (95% CI 11.1-41.8%) in the stop irinotecan arm (p=0.999). Similarly there was no difference in 1-year survival being 46.3% (95% CI 25.1-65.1%) in the continue irinotecan arm and 54.8% (95% CI 34.2-71.4%) in the stop irinotecan arm (p=0.11). Global quality of life scores 12 weeks after randomisation were not significantly different in the two groups (p=0.446).

Following 8 cycles of irinotecan the majority of patients have stopped treatment either due to PD or unacceptable toxicity. However, for the 16.5% of patients still benefiting from irinotecan there appears little advantage continuing beyond 8 cycles.

### P125

CAPECITABINEAND MITOMYCIN C (MMC) IS AN ACTIVE REGIMEN FOR PATIENTS WITH METASTATIC COLORECTAL CARCINOMA (MCRC) RESISTANT TO 5FU AND IRINOTECAN

S Rao*, D Cunningham, P.J Ross, T Price, C Ward, A.R Norman, M.E Hill
Royal Marsden Hospital, Downs Road, Sutton, Surrey SM2 5PT

Introduction: 5FU and MMC has demonstrated superior efficacy compared to infused 5FU alone in MCRC. MMC up regulates the expression of thymidine phosphorylase and thus capecitabine in combination with MMC may produce greater antitumour activity.
Objectives: To evaluate the safety and efficacy of capecitabine/MMC in MCRC resistant to 5FU and irinotecan.
Methods: An optimal 2-stage design was employed. Patients were required to have WHO performance status (PS)0-2, to be able to take oral medications, and to have adequate haematological, renal and hepatic function. All patients demonstrated progressive disease on chemotherapy or within 6 months of cessation. Capecitabine (1250mg/m$^2$ orally) was administered twice daily for 14 days followed by 7 days rest, every 3weeks, and MMC (7mg/m$^2$ IV bolus) was given every 6 weeks. CT response assessment according to RECIST criteria took place at 12 and 24 weeks.
Results: 31 patients have been accrued.17(55%) were male, median age was 64 (range 40-77) years, and 23 (74%) had PS0-1. 6 (19%) received 5FU based adjuvant therapy and 2 (6%) had prior radiotherapy. Sites of metastatic disease were liver 74%, lung 16%, peritoneum 16%, and nodal 12%. Of the 23 patients evaluable for response thus far, the ORR was 22% (95% CI: 6.8 -40.7) and13 (57%) had stable disease. There were no grade 4 toxicities; grade 3 toxicitiesinclude hand-foot syndrome 23%, diarrhoea 3.9%,vomiting 11.5% and neutropenia 3.7% Symptomatic improvement was as follows: pain 85%, dypsnoea 100% andbowel 86%.
Conclusion: Capecitabine and MMC is an active, well -toleratedregimen and is a valuable treatment option in MCRC refractory to 5FU and irinotecan.

### P126

ESTIMATION OF PLASMA THYMIDINE IN HEALTHY VOLUNTEERS VS. CANCER PATIENTS BY HIGH PERFORMANCE LIQUID CHROMATOGRAPHY (HPLC)

Tim Benepal*, Fraser Mitchell, Isobel Gibbens, Martin Gore and Ann Jackman. The Institute of Cancer Research, Sutton, Surrey.

Two pathways exist for the formation of thymidylate; a) the *de novo* pathway with thymidylate synthase (TS) being a key step, and, b) the salvage pathway in which thymidine (dThd) is the substrate. The latter has the potential to modulate the efficacy/toxicity of TS inhibitors such as 5-fluorouracil, methotrexate and the more specific inhibitors, raltitrexed and ZD9331. Human dThd levels have been reported to range from 0.1 to 7.4 μM (Holden *et al*, EJC, 1980) using radioimmunoassay, and to be higher in patients with haematological malignancies. Later, an HPLC technique quantified human and mouse levels of dThd to be <0.1μM and ~1μM respectively (Jackman *et al*, Biochem. Pharmacol, 1984). We have developed now a highly sensitive HPLC technique to accurately measure dThd. Plasma vs. serum dThd levels in 10 volunteers was compared with serum levels in 7 patients with ovarian cancer. In addition, we evaluated the possibility of thymidine phosphorylase (TP) released from platelets artifactually lowering sample dThd levels, by addition of a TP inhibitor to paired samples from 7 volunteers. Mean plasma vs. serum values in the volunteers was 14nM (4.5-25) vs. 12nM (4.5-23, p=0.57). Patient serum levels were lower than volunteers with a mean of 5.1nM (4-8, p=0.007). Addition of the TP inhibitor made no significant difference to results (-TP = 22nM range 5-48; +TP = 19nM, range 5-47, p=0.47). We conclude that the normal range of plasma/serum dThd in healthy volunteers is 5 to 50 nM. Patients with ovarian cancer have lower serum dThd levels. Future studies should evaluate the impact of pre-treatment dThd levels on the efficacy/toxicity of TS inhibitors in relevant tumour types. *Supported by CRUK.*

© 2003 Cancer Research UK

**P127**

ACUTE AND LATE TOXICITY FOLLOWING CONCURRENT WEEKLY CISPLATIN AND RADIOTHERAPY FOR CERVICAL CARCINOMA

Margaret King [1*], Chris McConkey [2], Andrew Hartley[1], Indy Fernando[1]
[1]Cancer Centre, Queen Elizabeth Hospital, Birmingham B15 2TH
[2]Cancer Research UK Clinical Trials Unit, University of Birmingham

**Introduction** A recent meta-analysis has shown a survival advantage for the addition of concurrent chemotherapy to radiotherapy in the treatment of cervical carcinoma. Controversy persists as to the potential magnitude of benefit and toxicity. A single centre experience is presented.

**Methods** All patients treated with concurrent chemo-radiotherapy from the 1st January 1999 to the 1st May 2002 were identified. Acute and late complications were scored using the NCI Common Toxicity Criteria and RTOG/EORTC system respectively. Univariate and multivariate analysis were performed to examine the relationship between demographics, stage, overall treatment time, radiotherapy dose, selectron insertion , number of chemotherapy cycles and occurrence of acute and late toxicity.

**Results** 79 patients received concurrent weekly cisplatin (40mg/m$^2$) with radiotherapy. Survival rate at 12 months was 91% (95% confidence interval 84-98%). At a median follow up of 18 months: 27 (34.2%) patients experienced 45 episodes of acute grade 3/4 complications and 9 patients (11.7%) experienced 16 late grade 3/4 complications. There was a significant correlation between grade3/4 acute toxicity and subsequent late grade 3/4 toxicity (p =0.002).

**Conclusions** Weekly cisplatin 40mg/m$^2$ concurrent with radiotherapy is well tolerated. However, severe acute toxicity carries a higher risk of subsequent late effects raising the possibility of consequential damage.

**P128**

MALIGNANT MIXED MESODERMAL TUMOURS OF THE OVARY: A RETROSPECTIVE REVIEW

Ewan Brown*, Moira Stewart, Tzyvia Rye, Awatif Al-Nafussi, Alistair R Williams, John Smyth, Hani Gabra
Cancer Research UK, University of Edinburgh Cancer Research Centre, Crewe Road South, Edinburgh, UK.

**Background:** Malignant Mixed Mesodermal Tumours (MMMTs) are relatively rare epithelial tumours that consist of both a carcinomatous and sarcomatous component.

**Methods:** Between 1984-2002, 69 patients with ovarian MMMT were referred to the Edinburgh Cancer Centre and variables including age, performance status, CA125 level, tumour stage, histological sub-type and type of treatment received were prospectively recorded. Outcome measures analysed were response to chemotherapy and overall survival.

**Results:** Patients had a median age of 67 (range 48-84). The majority of patients had advanced stage of disease at presentation (78% stage III/IV). Response rate (radiological and CA125 response) for first-line chemotherapy was 45% (10/22 patients). Median survival of the entire series was 8.9 months with a 1, 2 and 5 year survival rate of 44%, 24%, and 13% respectively. Stage III patients who were optimally debulked had a median survival of 12.1 months compared with 3.1 months for those who were sub-optimally or non-debulked. Responders to chemotherapy had a median survival of 19.4 months compared to 5.5 months for non-reponders (p=0.0115).

**Conclusion**: Ovarian MMMT is a distinct entity with poor prognosis. Achieving optimal debulking at initial surgery is an important factor in determining outcome and patients who respond to chemotherapy have a significant survival benefit. This analysis supports a role for chemotherapy and a need for randomised trials in ovarian MMMT.

**P129**

VAN DER BURG SCHEDULE (VDBS) IN RELAPSED EPITHELIAL OVARIAN CANCER (REOC):CAMBRIDGE ONCOLOGY CENTRE EXPERIENCE. Jennie Pratt, Bristi Basu, Cheryl Palmer, Mahesh Iddawela, Jean Abraham, Swethajit Biswas, James Brenton, Helena Earl*. Dept of Oncology, Addenbrookes Hospital, Cambridge CB2 2QQ

**Introduction:** VDBS in REOC has been reported as tolerable with high response rates (RR) and acceptable toxicity. **Aims:** Evaluate RR and tolerability of VDBS in patients (pts) with REOC in Cambridge. **Methods:** 19 pts with REOC were treated with VDBS. Cisplatin dose tailored to prior cisplatin exposure: prior cisplatin (PC) 60mg/m$^2$, wk 1-3: no prior cisplatin (NPC) 60mg/m$^2$, wk 1-3, and 70mg/m$^2$, wk 5-7. Oral etoposide dose 50mg/d wk 1-2 and 5-6. Dose intensity (DI) (dose delivered/dose planned) calculated. Response assessed by CT scan and Ca125. Toxicity assessed by CTC. Responding pts proceeded to maintenance oral etoposide (ME) (50mg/m$^2$/d 3wks x 6-9 cycles). **Results:** Median no. prior regimens was 2 (range 1-5), median treatment-free-interval (TFI) prior to VDBS was 5m (range 0.25-17m). 95% had prior taxanes;100% carboplatin;21% cisplatin. DI was 86% and 83% in PC and NPC groups respectively. A median of 3 cycles(range0-8) of ME was given. G3/4 toxicity: leucopenia(47%), fatigue(58%), anorexia(32%), infection(16%), diarrhoea(16%), N&V(58%). 3 deaths on induction treatment: neutropenic sepsis(1), bowel obstruction(1) and PE(1). Treatment stopped, delayed or dose-reduced due to toxicity in 84%. Following induction therapy radiological RR (CR/PR) was 64%, SD 27%, and Ca125 RR in those evaluable was 100%. Median progression-free-survival (PFS) from start of induction therapy was 6m on Ca125 and 7.75m on CT scan. **Conclusions:** VDBS in standard practice produced good RR and median PFS, but a high rate of toxicities, which should be taken into account given the palliative aim of treatment for pts with REOC.

**P130**

A PHASE II TRIAL OF ETANERCEPT, A TUMOUR NECROSIS FACTOR- α INHIBITOR IN RECURRENT OVARIAN CANCER

Sethupathi Ramalingam*, Susan Hoare, Srinivasan Madhusudan, Jeremy Braybrooke, Kulwinder Kaur, Sue Willner, Adrian L.Harris, Francis Balkwill, Trivadi S.Ganesan. CR UK Medical Oncology Unit, Churchill Hospital, Oxford OX3 7LJ, UK

TNF-α is strongly expressed in ovarian cancer. We have conducted a phase II trial of etanercept, a TNF-α inhibitor in patients (pts) with recurrent ovarian cancer. Initially, 17 pts (cohort I) received etanercept (25mg SC twice weekly) and subsequently 13 pts (cohort II) were treated at 25mg SC thrice weekly. All pts were planned to receive a minimum of 3 months of treatment up to maximum of 12 months. Disease response was evaluated at 3 monthly intervals. Surrogate end points for biological effects of etanercept included measurement of levels of TNF-α, TNF-R1, sE-Selectin, MCP-1, MMP-3 in plasma and whole blood cytokine release inhibition assay for IL-6 and MCP-1. The time points were pre-treatment, 24hours, 7 days, 28 days and 4 weekly there after. 18/30 pts have completed ≥ 3 months of treatment. There was no significant toxicity. 4/25 evaluable pts achieved stable disease at 3 months, which was maintained in 3 at 6 months. In cohort I, 9/13 pts had a significant reduction in IL-6 levels on day 1 which was maintained until 12 weeks (6/8). MCP-1 levels significantly declined in 9/13 pts on day 1 and by 3 months was inhibited by 50% (6/6). All other surrogate markers did not change with treatment. Definite biological effect was seen with 3 months of etanercept therapy. The trial is ongoing in cohort II pts receiving etanercept 25 mg thrice weekly.

© 2003 Cancer Research UK

**P131**

FEULGEN-LIGHT GREEN STAINING FOR CERVICAL SMEARS

J. Stephen *, Anitha B, J Sreeja, TKG Nair, VG Chellam.
* 'Beth-Shalom', TC 11/1326, Bains Compound, Nanthencode, Trivandrum 695003, INDIA. e-mail: jstephen@vsnl.com

Aim: To develop an alternative method for cervical smear staining.
Procedure: Cervical smears were made from 60 clinically suspected cases of carcinoma for the outpatients of Government SAT Hospital, Trivandrum, India together with those from ten healthy women as controls. Duplicate slides were made from each individual, one set stained by Papanicolaou method, the other by Feulgen light green method and examined under Leitz research microscope.
Major findings: Feulgen-light green staining was found to be superior to Papanicolaou staining in revealing finer details of nuclei and chromosomes which stained bright magenta in a light green cytoplasmic background. The light green Feulgen stain showed better affinity to cancer and precancer cells than to normal cervical cells. The study also revealed that the presence of micronuclei and more than one Barr body per nucleus is associated with cervical carcinoma progression.
Significance: Feulgen stained preparations can be used in automated DNA determination per nucleus by microspectrophotometry and can be employed in rapid screening for carcinoma of the cervix.
Conclusion: Feulgen-light green staining is a superior and sensitive method to detect carcinoma of the cervix.
References:

Feulgen, R; Rossenbeck, H 1924. Z.Physiol.Chem. 135:203
Papanicolaou GN. 1942. Science 95:438

**P132**

CARBOPLATIN (C) FOLLOWED BY SEQUENTIAL WEEKLY PACLITAXEL (T) AND GEMCITABINE (G) AS FIRST-LINE TREATMENT FOR OVARIAN CANCER.

M. Harries*, C. Moss, T. Perren, M. Gore, G. Hall, M. Everard, R. A'Hern, C. Cole, I. Gibbens, A. Jenkins and S. Kaye. Royal Marsden Hospital.

**Objectives**: We evaluated the feasibility of delivering C followed by sequential weekly T and G in patients with advanced, previously untreated ovarian cancer.
**Methods**: Women with FIGO stage Ic-IV ovarian cancer were treated with 4 cycles of C AUC 7 q21 followed by 4 cycles of T 70 mg/m$^2$ (d1, 8, 15) and G 1000 mg/m$^2$ (d1, 8) q21.
**Results**: 53 patients enrolled, with a median age of 59 years (range, 29-77). 37 (70%) patients had FIGO stage III/IV and 22 (42%) had residual disease >2 cm. 29 (55%) patients completed all planned cycles of treatment. 24 patients stopped early, including 4 with progressive disease and 7 with pulmonary toxicity. Of 370 total cycles of chemotherapy delivered, 27 cycles required a dose reduction and 68 cycles were delayed, primarily due to myelosuppression, during the weekly phase. Of the 26 patients with radiologically evaluable disease, 23 (88%) had partial or complete responses. Of 36 patients with an elevated CA-125 at baseline, 30 (83%) had a decrease ≥75%. CTC grade 3/4 hematological toxicity, predominantly neutropenia, was seen in 58% of cycles. The most significant non-hematological toxicity was pulmonary, which was reported in 7 (13%) patients with breathlessness, decreased transfer factor +/- radiological interstitial lung changes during the weekly phase of treatment. This toxicity was reversible and required steroids in only 2 patients. No significant neurotoxicity was seen.
**Conclusions**: This regimen is generally well tolerated and associated with an acceptable response rate. However, the level of pulmonary toxicity, which may be a feature of the weekly taxane-G regimen, was of some concern. Thus, alternative schedules, including three-weekly taxanes, are currently being evaluated.

**P133**

A PHASE II TRIAL OF CAPECITABINE AND MITOMYCIN C (MMC) IN 1st LINE TREATMENT OF METASTATIC COLORECTAL CANCER (MCRC).

S Rao*, D Cunningham, P.J Ross, T Price, D Watkins, A.R Norman, P Shellito, M.E Hill
Royal Marsden Hospital, Downs Road, Sutton, Surrey SM2 5PT

**Background:** The combination of Mitomycin C (MMC) and infused 5FU has been shown to have greater efficacy than infused 5FU alone in the treatment of MCRC. MMC is known to upregulation intratumoural thymidine phosphorylase (TP) expression. The upregulation of intratumoural TP by MMC may be exploited by the use of MMC in combination with the oral fluoropyrimidine capecitabine. This phase II study evaluates the efficacy and tolerability of MMC and capecitabine combination as initial therapy in MCRC.
**Methods:** Eligibility; no prior chemotherapy for advanced disease or adjuvant therapy in previous 6 months, measurable disease, ECOG performance status (PS) ≤ 2. Patients (pts) received MMC 7mg/m$^2$ IV bolus every 6 weeks and oral capecitabine 2500mg/m$^2$/day given for 14 days followed by 7 days rest (every 3 weeks), pts completed a total of 24 weeks of therapy. CT response assessment was performed every 12 weeks according to RECIST criteria.
**Results:** Toxicity data is available on 46 pts and response data on 44 pts who have completed >12 weeks of treatment. Median age 70 (range 30-82) years, 67% of pts had ≥ 2 sites of disease. Objective response rate is 45.4% (95% CI; 30.4%-61.1%) in evaluable pts, stable disease was seen in a further 26.6%. Median failure free survival is 7.6 months. No grade 4 toxicity occurred, grade 3 toxicities include; hand-foot syndrome 24%, diarrhoea 11%, neutropenia 4%, nausea and vomiting 2.2%.
**Conclusion:** Capecitabine /MMC appears to show promising activity in MCRC with an acceptable toxicity profile and a convenient administration schedule.

**P134**

QUALITY OF LIFE AFTER 5YEARS TAMOXIFEN FOR WOMEN WITH EARLY BREAST CANCER.

P Hall, J Walkington, S Douglas, L Renshaw, D A Cameron Edinburgh Breast Unit, Western General Hospital, EDINBURGH EH4 2XU

With no clear evidence suggesting a breast cancer benefit for continuing tamoxifen beyond 5 years, we were interested in the effect of stopping tamoxifen on patients' quality of life (QoL). With local ethics approval, patients identified as being disease free and having completed 5 years' tamoxifen patients were recruited. After giving written consent, patients completed the EORTC QLQ C30, together with supplementary questions relating to other possible tamoxifen side effects. Three months after stopping their tamoxifen, they were sent the same questionnaires to be returned by post. 46 have completed both questionnaires, with an average age of 61. Baseline QoL was similar to normal populations. Three months later there was no overall change in QoL for these women, with most items showing no change, apart from:

| Mean | On tamoxifen | 3 months later | Change? |
|---|---|---|---|
| Global QoL | 79.5 | 76.6 | NS |
| **Role Function** | **92** | **88** | **p = 0.05** |
| **Social Function** | **93.7** | **90.6** | **p = 0.01** |
| **Fatigue** | **14.4** | **20.6** | **p = 0.003** |
| Median score (1 – 7) | | | |
| **Nausea/Vomiting** | **4.7** | **2.2** | **p = 0.05** |

These data suggest that there is a low level of GI toxicity associated with tamoxifen, and some loss of social functioning associated with stopping it. Larger studies are required to see if there is a subgroup of patients who need additional support after stopping tamoxifen.

© 2003 Cancer Research UK

**P135**

BAG-1 POTENTIATES OESTROGEN DEPENDENT TRANSCRIPTION AND PROTECTS BREAST CANCER CELLS FROM STRESS-INDUCED GROWTH INHIBITION

Graham Packham*, RamseyI Cutress, Paul A Townsend, AdamSharp and Peter WM Johnson.
Cancer Research UK Oncology Unit, Cancer Sciences Division, School of Medicine, Universityof Southampton, Southampton General Hospital, Southampton, SO16 6YD.

BAG-1 is a multifunctional protein which exists as three isoforms, BAG-1S, BAG-1M and BAG-1L. BAG-1 proteins interact with a diverse array of cellular targets and regulate a wide range of cell growth control pathways including survival, proliferation and the function of some nuclear hormone receptors. Overexpression of BAG-1 has been described in breast cancer where it may predict clinical outcome. However, the functional significance of BAG-1 overexpression has not been studied in detail. Overexpression of BAG-1 has profound effects on the biology of breast cancer cells. BAG-1L, which resides in the nucleus, interacted with oestrogen receptor alpha and stimulated oestrogen dependent transcription. All BAG-1 isoforms protected breast cancer cells from apoptosis and long term growth inhibition induced by cellular stress. Suppression of stress-induced growth inhibition required a conserved lysine in the BAG-1 N-terminal ubiquitin-like domain and C-terminus residues important for interaction with 70 kDa heat shock proteins, HSC70 and HSP70. Therefore, overexpression of BAG-1 is likely to play an important role in breast cancer and targeting key protein:protein interactions required for its function may be an attractive strategy to counter its activity.

**P136**

VITAMIN D RECEPTOR SIGNALLING IS CORRUPTED IN BREAST CANCER CELLS VIA A HISTONE DEACETYLATION MECHANSIM

Claire M. Banwell, Laura P. O'Neill & Moray J. Campbell; Division of Immunity and Infection, Medical Sciences, Univ. Birmingham, B15 2TH

The intrinsic ability of 1α,25 dihydroxyvitamin $D_3$ (1α,25$(OH)_2D_3$) to control the proliferation of breast epithelial cells is corrupted in malignancy thus impeding its clinical application. We hypothesised that epigenetic mechanisms suppress antiproliferative target genes in 1α,25$(OH)_2D_3$-insensitive breast cancer cells. In support of this we found that resistance to the antiproliferative actions, in a range of malignant breast cell lines (T-47D, ZR-75-1, MCF-7, MCF-$7_{Res}$ and MDA-MB-231) and the non-malignant breast epithelial cells MCF-12A cells, correlated with significantly reduced vitamin D receptor (VDR) and increased nuclear receptor co-repressor mRNA.

Subsequently we demonstrated that the histone deacetylation inhibitors sodium butyrate (NaB) and trichostatin A (TSA) showed strong additive and synergistic antiproliferative effects when combined with 1α,25$(OH)_2D_3$ or its potent analogs that were resistant to metabolism. For example, after 96 hours, 1,25-Dihydroxy-16,23,Z-diene-26,27-hexafluoro-19-nor vitamin $D_3$ (RO-26-2198) (100nM) alone inhibited MDA-MB-231 cells by 13%, TSA (15nM) resulted in 26%, and the combination resulted in 61% inhibition ($p$<0.001). Reflective of this the co-treatment alone resulted in a significant $G_1$ cell cycle arrest after 24 hours. Consistent with the hypothesis of repressed antiproliferative target genes, we undertook real time RT-PCR time course studies to confirm that the co-treatment of agents uniquely modulated antiproliferative targets such as the CDKI p21$^{(waf1/cip1)}$ and the metastasis suppression gene VDUP-1. Furthermore preliminary data suggests that the co-treatment of agents sustains bulk histone acetylation. Thus combined chemotherapy with 1α,25$(OH)_2D_3$ analogs and clinically relevant HDAC inhibitors may deliver therapeutic regimes, which overcome toxic side-effects and sustain anticancer effects.

**P137**

CHARACTERISATION OF DOCETAXEL RESISTANT BREAST CANCER CELL LINES

Sarah L. McDonald*, Susan E. Moir, Ahmed O. Babalghith, David A. J.Stevenson, Steven D. Heys, Heather M. Wallace, Andrew. C. Schofield. Depts. of Surgery, Molecular and Cell Biologyand Medicine and Therapeutics. University of Aberdeen, AB25 2ZD. Scotland.

Docetaxel is one of the most effective chemotherapy agents used to treat advanced breast cancer. Unfortunately, many patients develop resistance to treatment and the resistance pathways are largely unknown. In order to investigate the mechanisms involved we have developed docetaxel resistant MCF-7 and MDA-MB-231 cell lines. The aims of the project were to characterise the resistant cell lines and to identify novel regions of genomic change involved in resistance to docetaxel using Comparative Genomic Hybridisation (CGH).

The docetaxel resistant cell lines were generated by sequential exposure to docetaxel. Resistance was demonstrated by MTT assay. Genomic changes between the parental cells and the resistant sub-line was investigated by CGH. Western analysis was used to demonstrate changes in protein expression.

Exposure to increasing concentrations of docetaxel for 50 weeks resulted in cells able to withstand 24 h exposure of 30 μM docetaxel. Western analysis revealed that the resistant cells overexpress P-glycoprotein. In addition, CGH showed a large amplification of 7q21.3-7q31.1 in the MCF-7 resistant subline.

This is the first description of the use of CGH to identify genes involved in docetaxel resistance. Further investigation of these cell lines will lead to a better understanding of the mechanisms of resistance and ultimately may result in the development of more effective therapy.

**P138**

BCL-2 OVEREXPRESSION IN A DOCETAXEL RESISTANT BREAST CANCER CELL LINE.

Iain Brown*, Sarah L McDonald, Susan E Moir, Steven D Heys, Andrew C Schofield.
Dept. of Surgery, Medical School, University of Aberdeen.

**Introduction**: Docetaxel is the most effective single agent regimen in advanced breast cancer therapy. However, up to 50% of patients are resistant to the disease. There may be many reasons why a tumour may be resistant to chemotherapy including innate and acquired resistance mechanisms.

**Aims:** There is limited data on molecular factors involved in resistance to docetaxel. We sought to determine, therefore, the molecular changes in breast cancer cell lines which had been made resistant to docetaxel in order to further understand the mechanisms of resistance to this drug.

**Methods:** MCF-7 cells were made resistant to docetaxel by continual exposure to increasing amounts of the drug over a period of time. We looked at the levels of expression of proteins expected to be involved in apoptosis or resistance (P-glycoprotein, bcl-2, bax, p53, mdm2, p16) by western analysis in resistant and wild-type cells.

**Results:** Docetaxel resistant cells overexpressed bcl-2 and p-glycoprotein compared to wild-type cells which had no detectable levels of each protein. p16, bax and p53 levels were unchanged. No mdm2 was detected in either cell line.

**Significance and conclusion:** It is known that bcl-2 expression is upregulated in response to docetaxel treatment, but this is the first time it has been shown to be permanently upregulated in a resistant cell line. It is likely that there is more than one mechanism of resistance other than p-glycoprotein overexpression in these cells and we are currently looking at other apoptosis, cell cycle, and drug metabolism pathways, in these and another resistant cell line, to further understand these mechanisms.

© 2003 Cancer Research UK

**P139**

DIINDOLYLMETHANE INDUCES APOPTOSIS AND INHIBITS PKB/AKT PHOSPHORYLATION IN THE MDA MB468 BREAST TUMOUR CELLLINE

Mark J Anderton*, Lynne M Howells, E Ann Hudson, William P Steward, Margaret M Manson, Biocentre and Dept. of Oncology, University of Leicester, UK

Diindolylmethane (DIM) is a major *in vivo* derivative of the anticancer agent indole-3-carbinol (I3C). We showed previously that I3C induces apoptosis in the MDA MB468 breast tumour cell line but not in the normal breast tissue derived HBL100 line. This effect was in part due to the ability of I3C to inhibit phosphorylation and activity of protein kinase B (PKB/Akt) in the MDA MB468 cell line only. In the present study we compared the ability of DIM with that of I3C to inhibit cell growth and induce apoptosis in these two cell lines. DIM was a more potent inhibitor of cell growth with approximate $IC_{50}$ values (at 168 hr) in MDA MB468 and HBL100 cell lines of 5 and 40 µM respectively, compared to 30 and 120 µM for I3C. Like I3C, DIM was able to induce apoptosis in the MDA MB468 cell line as determined by annexin V staining, but again showed greater potency, with 50 µM DIM causing a similar degree of apoptosis as 200 µM I3C at 24 and 48 hrs (10 and 45 % respectively). Using Western blotting, DIM was shown to inhibit phosphorylation of PKB, more quickly and with a higher potency than I3C. After 5 hrs, 100 µM DIM inhibited PKB phosphorylation by approximately 50%, a degree of inhibition that required 500 µM I3C. Neither compound affected total PKB protein levels. Inhibition of PI3K/PKB signalling may therefore be an important chemopreventive mechanism of DIM. In conclusion, DIM showed considerably greater potency and more rapid activity than its parent compound, and is an exciting prospect as a chemopreventive agent.

**P140**

EXPRESSION OF HUMAN DELTA-6-DESATURASE AND DELTA-4-DESATURASE IN HUMAN BREAST CANCER AND THE ASSOCIATION WITH CLINICAL OUTCOMES.

Jane Lane, Robert E. Mansel, Wen G. Jiang. Metastasis Research Group. University Department of Surgery, University of Wales College of Medicine, Heath Park, Cardiff, The United Kingdom

Highly unsaturated fatty acids (HUFAs) are cytotoxic to cancer cells and generated in the body by desaturation from essential fatty acids such as alpha-linolenic acid and linoleic acid. The desaturation is primarily mediated delta-6-desaturase. This study, for the first time, examined the level of expression of human delta-6-desaturase as well as delta-4-desaturase in human breast cancer. Human breast tumours (n=102) which comprised of 88 ductal and 14 lobular carcinomas, as well as normal breast tissues (n=31), together with breast cancer cell lines were analysed for the level of expression of delta-6- and delta-4-desaturases using RT-PCR and quantitative PCR. A lower level of delta-6-desaturase was seen in breast tumour compared with normal tissues. Tumours from patients who had a poor prognostic index and from those who died of breast cancer had the lowest level of delta-6-desaturase (median follow-up 72 months). In addition, TNM3 and TNM4 tumours had significantly lower level of delta-6-desaturase than TNM1 tumours. Interestingly, ductal tumours displayed significantly higher level of the enzyme than lobular tumours. In contrast, a stepwise increase of delta-4-desaturase was seen in tumour from patients with poor prognosis. It is concluded that aggressive breast tumours have a reduced level of delta-6-desaturase. This aberrant expression has clinical bearings to the outcome in patients with breast cancer.

**P141**

DETERMINATION OF MICROVESSELS IN HUMAN BREAST CANCER USING VASCULAR ENDOTHELIAL CADHERIN (VE-CADHERIN)

Tracey A. Martin*, Gareth Watkins, Jane Lane, Wen Guo Jiang. Metastasis Research Group, Department of Surgery, UWCM, Cardiff, CF14 4XN.

VE-cadherin is a calcium-dependent endothelial-specific cell adhesion molecule associated with adherens junctions. Cadherin expression is believed to facilitate morphogenic events such as migration, proliferation, and differentiation.

This study aimed to determine if VE-cadherin could be used as a suitable marker to assess angiogenesis and whether a correlation exists between levels of VE-cadherin and angiogenic markers Factor-8 & PECAM-1 with patient outcome in breast cancer. Frozen sections from breast cancer primary tumours (matched tumour n=114, & background n=30) were immuno-stained with VE-cadherin, Factor-8 and PECAM-1 antibodies and microvessel number assessed. RNA from the tissues was reverse transcribed and quantified before analysis by Q-PCR. VE-cadherin, Factor-8 and PECAM-1 were targeted as angiogenic markers. Results were expressed as copy numbers of transcript/50 ng RNA (β-actin standardised).

Immunohistochemical staining for all three markers showed a significant difference in microvessel number in tumour sections compared to background with VE-cadherin (background: 7.29 +/- 0.77 vessels vs tumour 17.56 +/- 1.5, p=0.0008). There was no significant difference in the number of microvessel stained with either PECAM-1 (background 2.08+/-0.78 vs tumour 2.36+/-0.25, p=0.75) or Factor-8 (background 59.8+/-12 vs tumour 49.4+/-4.7, p=0.43) where there was increased staining of other structures within the sample and higher background. In addition, Q-PCR showed elevated levels of VE-cadherin and PECAM-1 in tumour samples compared to background tissue (VE-cadherin: tumour 2.356+/-0.64 vs background 1.979+/-0.613 p=0.7; PECAM-1: tumour 275+/-73.7 vs background 145.7+/-30 p=0.8). These markers were also elevated in patients with poor prognosis, as determined by NPI status (VE-cadherin: NPI3 7.3+/-4.8 vs NPI1 2.25+/-0.697; PECAM-1: NPI3 814+/-519 vs NPI1 256.2+/-89.2). There was no difference in levels with Factor-8.

We conclude that higher levels of angiogenic marker molecules in breast cancer are associated with poor prognosis in patients. Moreover, VE-cadherin appears to be a preferable marker for such analysis.

**P142**

DEVELOPMENT AND CHARACTERISATION OF AN *IN VITRO* CELL MODEL OF TAMOXIFEN-RESISTANCE.

Alicia Parkes*, Sarah Burdall, and Valerie Speirs. Molecular Medicine Unit, University of Leeds, Leeds, LS9 7TF.

Tamoxifen (TAM) is an established adjuvant treatment for hormone-dependent breast cancer and more recently in the prevention of the disease in high-risk individuals. Despite its success, the majority of initially responsive patients (60-70%) eventually relapse. The aim of this study was to develop and characterise an *in vitro* model of resistance using the TAM-sensitive breast cancer cell line MCF-7, by continuous culture in 0.1µM 4-Hydroxy-Tamoxifen (4-HT) over 28 months. Cell phenotype and response to ER ligands was monitored as resistance developed, on a monthly basis. Cytogenetic analysis is on-going. wtMCF-7 express ERα, -β, and PR and were initially inhibited by 4-HT by 50% over controls. By flow cytometry, cells in S-phase were also significantly reduced. By month 4 the inhibitory effect of TAM was lost, indicating the acquisition of resistance (MCF-7r). This effect was consistent up to 28 months. In comparison to wtMCF-7, MCF-7r grew more slowly (30% less than wt). Upon removal of TAM, the resistant phenotype was retained, although addition of 0.1nM 17β-estradiol (E2) had an agonistic effect. In the continued presence of TAM, the effect of E2 was concentration-dependent: agonistic at 0.1nM E2, but antagonistic below 0.01nM, suggesting a competitive interaction between the two compounds. ERα, -β and PR were retained in MCF-7r indicating a functional ER-signalling pathway. Using a clonogenic assay, 6 individual clones were isolated from MCF-7r. These retain resistance to TAM but show differences in morphology and *in vitro* growth. This cell line model will be valuable in understanding the mechanisms involved in the acquisition of TAM-resistance.

© 2003 Cancer Research UK

### P143

A PHASE 2 STUDY OF CHLORAMBUCIL AND LOMUSTINE (CL56) IN THE TREATMENT OF ABSOLUTE HORMONE REFRACTORY PROSTATE CANCER

Gairin Dancey*, Jonathan Shamash, Pompa Bhattacharyya, Peter D. Wilson, Lynda J. Millard and R.Timothy D.Oliver. Department of Medical Oncology, St Bartholomew's Hospital, London.

The last two years has seen an increasing interest in the use of chemotherapy for prostate cancer using combinations of estramustine and iv docetaxel. In this elderly population the potential benefit of a simple oral regimen was explored. 37 patients (33 symptomatic) with a median age of 71 and performance status of 0-3 were enrolled. All patients had previously had second line hormonal therapy with a steroid and an oestrogen. On progression, hormones were withdrawn and chlorambucil 1mg/kg, given as 6mg a day until total dose reached and lomustine 2mg/kg on day 1 were given and repeated every 56 days (CL56).

**Results**:1 patient normalised his PSA and 3 had > 50% decline to give a PSA response rate of 10%. 65% achieved disease stabilisation beyond 8 weeks and 54% had a symptomatic response. The median time to progression was 3.6 months (4.6 months in those with ≥ SD, vs 1.2 months in those with PD, $p<0.05$) with an overall survival of 7.1 months. Toxicity was mild with grade 3 toxicity of malaise 14% and constipation 12%. The median survival from becoming androgen independent was 23.5 months. 4/15 who progressed on chemotherapy responded again to hormonal manipulation when rechallenged. It is concluded that the sequential use of androgen deprivation, followed by steroids and oestrogens and then chlorambucil and lomustine leads to comparable survival to the current estramustine and taxane based therapies.

### P144

NEP AND ECE CAN MODULATE STROMAL-EPITHELIAL INTERACTIONS AND CAN INFLUENCE INVASIVE BEHAVIOUR OF PROSTATE CANCER CELLS

Louise A Dawson*[1], Norman J Maitland[2], Anthony J Turner[1], Badar A Usmani[1]
[1]School of Biochemistry & Molecular Biology, University of Leeds, Leeds LS2 9JT; [2] YCR Unit, Dept. of Biology, University of York YO10 5YW

The structurally related metalloproteinases endothelin-converting-enzyme (ECE) and neprilysin (NEP) contribute respectively to the synthesis and inactivation of the mitogenic peptide endothelin-1 (ET-1). Changes in a developing tumour during stromal-epithelial interactions can contribute to cancer invasion and metastasis as a consequence of the availability of endothelin-1. This study has investigated the interaction between metastatic tumour epithelial cells which lack NEP and stromal cells which express ECE-1, using Matrigel invasion chambers. Data show that PC cell invasion through Matrigel is increased in the presence of ECE-1 expressing stromal cells. Inhibition of endogenous ECE-1 activity in stromal cells using an ECE specific inhibitor reduced PC-3 and Du145 invasion by ~70% and 50% respectively. Inactivation of mitogenic peptides by the addition of recombinant NEP to PC-3 and Du145 cells also reduced invasion by ~50% and 20% respectively. Alternatively, supplementation of defined media with bradykinin and ET-1 significantly increased PC-3 invasion by ~40% and 50% respectively. Du145 cell invasion increased 100% on the addition of ET-1. Transient expression of an ECE-1c isoform in PC-3 cells increased invasion above control by ~15%, whereas transient expression of ECE-1a and ECE-1b isoforms reduced invasion by 40% above control. ECE-1a and ECE-1b isoforms are routinely absent in metastatic cells. These studies reveal that stromal/epithelial interactions can influence the invasive ability of PC cells partly as a consequence of their relative NEP and ECE-1 activity and this effect may be attributed to the ECE-1c isoform in particular.

### P145

SERUM PROSTATE SPECIFIC ANTIGEN (PSA)-NEGATIVE METASTATIC PROSTATE CANCER:HISTOPATHOLOGY AND AN ALTERNATIVE DIAGNOSTIC MARKER.

Alison J.Birtle* Alex Freeman,Stephen J.Harland, Heather A.Payne & John R.M.Masters.Prostate Cancer Research Centre,67,Riding House St, London.W1W 7EJ.

Approximately 1% of men present with "PSA-Negative" prostate cancer (PCA), having serum PSA levels much lower than the tumour burden would suggest. Diagnosis and treatment can be delayed, and PSA levels are unreliable in monitoring disease. Alternative markers are needed for diagnosis and surveillance. We describe the histopathological features and marker expression of 14 prostate cancers from men presenting with metastatic disease and low PSA. Patients presenting with metastatic PCA, with serum PSA of less than 10 ng /ml were identified from the BAUS Cancer Registry. Formalin fixed, paraffin-embedded archival prostatic tissue was antigen retrieved and immunostained using monoclonal antibodies for PSA, prostate specific membrane antigen (PSMA), prostate specific acid phosphatase (PSAP), androgen receptor and neuroendocrine markers. Semi-quantitative scoring was performed.14 cases have been analysed. Despite low serum PSA levels, focal tissue staining for PSA was seen in 11/ 14 cancers. PSMA staining was observed in all cancers, with a diffuse pattern and was present in cancer cells negative for PSA in 9 / 14 cases. Androgen receptor staining was positive in 12/14 and PAP in 10/14 cases. 7 cases were weakly positive for neuroendocrine markers. Median Gleason score was 9. PSMA is diffusely present in prostatic tissue in serum "PSA-Negative" metastatic PCA patients. Focal tissue staining for PSA may easily be missed on biopsy. PSMA can aid histopathological diagnosis of prostate cancer in patients presenting with clinical signs of metastatic PCA and low serum PSA. Significant neuroendocrine differentiation was not a feature.

### P146

IS INCREASED LIPID PEROXIDATION IN PATIENTS WITH PROSTATE CANCER DUE THE SYSTEMIC INFLAMMATORY RESPONSE OR INSUFFICIENT INTAKE?

Ahmad Almushatat*, Donald C McMillan, Dinesh Talwar,Peter A McArdle, Naveed Sattar, Denis StJ O'Reilly, Mark A Underwood

**Introduction**

Oxidative stress is recognised to contribute to the nutritional decline of cancer patients. The basis of this oxidative stress could either be due to the tumour itself or a host response. The polyunsaturated fatty acid residues of phospholipids are extremely sensitive to oxidation. The major product of lipid peroxidation is malondialdehyde (MDA). To determine whether oxidative stress was due to the presence of cancer, the systemic inflammatory response or insufficient intake of antioxidants, we examined the relationship between lipid peroxidation (MDA), lipid soluble vitamins ($\alpha$-tocopherol and carotenoids), host systemic inflammatory response (C-reactive protein) and stage of disease in patients treated for prostate cancer.

**Patients and methods**

Patients with BPH (n=19), localised (n=32) and metastatic (n=39) prostate cancer were studied. Blood samples were removed for the measurement of PSA, CRP, MDA, cholesterol, $\alpha$-tocopherol, lutein, lycopene, $\alpha$–carotene, and $\beta$-carotene. MDA concentrations were adjusted for cholesterol. The study was approved by the local ethics committee.

**Results**

Increased MDA concentrations were associated with more advanced stage of disease and reduced lutein and lycopene concentrations. In contrast, there was no significant alteration in the other lipid soluble vitamins or C-reactive protein

**Conclusion**

Lipid peroxidation and reduced carotenoid concentrations appear to be dependent on stage of disease but not the low grade systemic inflammatory response in patients with prostatic disease. This would suggest that there is insufficient dietary intake of carotenoids for the oxidative stress associated with the presence of prostate cancer.

© 2003 Cancer Research UK

**P147**

DEXAMETHASONE AS AN ALTERNATIVE TO CORTISONE OR HYDROCORTISONE IN COMBINATION WITH STILBOESTROL FOR HORMONE REFRACTORY PROSTATE CANCER

Madhumita Bhattacharyya*, Garin Dancey, Peter Wilson, Wendy Ansell, Jonathan Shamash, Tim Oliver, J Ong, Medical Oncology, Barts and the London Medical School

**Introduction and Aim:**

Farrugia et al 1998 observed a high PSA response rate (78%) when Stilboestrol + hydrocortisone were given to patients with hormone refractory prostate cancer. Reports suggesting Dexamethasone was more effective than hydrocortisone as a single agent in these patients lead to a phase 2 study to evaluate Stilboestrol + Dexamethasone (S+D). This abstract reviews these results and compares them with updated results from use of Stilboestrol + other corticosteroids (S+C/HC).

**Procedure:**

M+ or M0 symptomatic with rising PSA on > 1 occasion while receiving primary endocrine treatment were selected. Stilboestrol 1mg, Dexamethasone 2mg + aspirin 75mg were given daily. In the previous protocol patients received hydrocortisone 20mg bd or cortisone acetate 25mg o'mane and 12.5mg o'nocte

**Results:**

73 patients treated with S+C/HC, 41 with S+D. Median time to going off study was 15.2 months after S+C/HC v 13.5 after S+D. At 2 years 16% on S+ C/HC and 20% on S+D remain on treatment. On progression, duration of response to Chlorambucil/Lomustine was 6.8 months in those who failed S+C/HC and 8.8 months S+D. 50% PSA response rate, was 73% on S+ C/HC and 78% on S+D. Though toxicity was similar, Dexamethasone produced more fluid retention.

**Conclusion:**

Despite the reported advantage of Dexamethasone over cortisone as single agent, there seems to be no major advantage of Dexamethasone when combined with Stilboestrol as an alternative to cortisone or hydrocortisone.

**P148**

ANDROGENS INCREASE OXIDATIVE DNA ADDUCT LEVELS IN HUMAN PROSTATE CELLS

SK Pathak*, RA Sharma, R Singh, PB Farmer, WP Steward, AJ Gescher, JK Mellon. Departments of Oncology, Urology & Biochemistry, University of Leicester, Leicester LE2 7LX, UK.

Reactive oxygen species involved in carcinogenesis have been shown to attack DNA bases. Two adducts arising from oxidative DNA damage are 8-oxo-deoxyguanosine (8-oxo-dG) and pyrimidopurinone adducts of deoxyguanosine ($M^1G$). Both adducts have been associated with mutagenesis and carcinogenesis. Dihydrotestosterone (DHT) causes oxidative stress in androgen-sensitive human prostate cancer cells, but its effect on oxidative DNA adducts has not been studied. We tested the hypothesis that DHT increases levels of oxidativeDNA adducts in androgen-sensitive LNCaP cells. We also studied its effect in androgen-insensitive PC3 and DU145 cells. 8-Oxo-dG levels were measured by LC-MS/MS with correction for amount of DNA by HPLC-UV, and $M^1G$ levels by immunoslot blot with correction by propidium iodide staining. Mean 8-oxo-dG levels in control LNCaP, PC3 and DU145 cells were 2.9, 3.5 and 4.1 adducts/106 nucleotides respectively, and $M^1G$ levels were 12.6, 6.9 and 7.5 adducts/$10^7$nucleotides respectively. $M^1G$ levels in DHT-treated LNCaP, PC3 and DU145 cells were 17.2, 7.9 and 7.4 adducts/107 nucleotides respectively, and 8-oxo-dG levels were 4.9 adducts/106 nucleotides in LNCaP cells. The difference in adduct levels between control and DHT-treated cells was significant only for $M^1G$ levels in LNCaP cells ($P < 0.05$ by ANOVA). The results represent the first report of measurable oxidative DNA adducts in human prostate cancer cells grown *in vitro* and suggest that androgens can increase M1G levels in androgen-sensitive cells.

Supported byUK MRC and Prostate Research Campaign UK.

**P149**

HEPATOCYTE GROWTH FACTOR/SCATTER FACTOR REGULATES THE EXPRESSION OF HGF ACTIVATION REGULATORS, HAI-1 AND MATRIPTASE-1, IN HUMAN PROSTATE CANCER CELLS.

Rhidian A. Hurle[1], Chris Parr[1], Spencer A. Jenkins[2], Howard G. Kynaston[2], Wen G. Jiang[1]. [1]Metastasis Research Group, University of Wales College of Medicine, and [2]Department of Urology, University Hospital of Wales, Cardiff CF14 4XN, UK.

Hepatocyte growth factor (HGF), otherwise known as scatter factor (SF), is a cytokine that plays an active role in the migration and invasion of cancer cells, including prostate cancer cells. This study examined the role of HGF on the expression of HGF activators, Matriptase-1 and HGFA, and HGF activation inhibitors HAI-1 and HAI-2 in human prostate cancer cells.

A non-invasive human prostate cancer cell line, CA-HPV-10 and a highly invasive line PC-3 were used in the study. Cancer cells were treated with recombinant human HGF for periods upto 24 hours. The mRNA levels of HAI-1, HAI-2, HGFA and matriptase-1 were determined using RT-PCR and quantitative PCR, with β-actin as control house keeping gene. The protein levels of these molecules were assessed using Western blotting from cells similarly treated with HGF. HGF increased the transcript levels of matriptase-1 in PC-3 cells (Threshold cycle No. 42.6±1.02 in control and 35.6±0.39 in HGF treated cells, p<0.01). The effect of HGF on matriptase-1 in CA-HPV-10 cells was, however, insignificant (Threshold cycle No. 37.9±0.32 in control and 38.3±1.4 in HGF treated cells, p=0.38). In contrast, HGF increased the levels of expression of HAI-1 in both cells, with the most profound effect seen in CA-HPV-10 cells (39.1±6.5 vs 34.7±1.6 in CA-HPV cells and 33.7±0.8 vs 32.4±0.3 in PC-3 cells). The effect of HGF on the levels of expression of HAI-2 was inconclusive. It was further revealed that the changes in mRNA of HAI-1 in prostate cancer cells were reflected at protein level. Western blotting showed an increased amount of HAI-1 protein in HGF-treated cells compared with untreated cells.

It is concluded that hepatocyte growth factor has direct effect on the molecules that regulates the activation of pro-HGF, such as HAI-1 and matriptase-1 and that HGF may have a wider role in its action on prostate cancer cells.

**P150**

POTENTIATION OF RADIOTHERAPY BY THE EGFR TYROSINE KINASE INHIBITOR ZD1839 (IRESSA) ON BLADDER CANCER *IN VITRO.*

Satish B. Maddineni*[1], Vijay Sangar[1], Geoff P. Margison[1], Jolyon H. Hendry[1], Kieran J. O'Flynn[2], Noel W. Clarke[2,3].

[1]Carcinogenesis Group, Paterson Institute for Cancer Research, Manchester, [2]Salford Royal Hospitals NHS Trust, Salford, [3]Christie Hospital NHS Trust, Manchester, UK.

**Introduction:** Radiotherapy is an established organ preserving radical treatment for invasive bladder cancer. Enhancing the response to ionising radiation (IR) may further improve prognosis. We established the radiosensitising properties of ZD1839 on 2 related bladder cancer cell lines of differing radiosensitivities.

**Methods:** The radiation survival curves for MGH-U1 (radioresistant) and its mutant clone S40b (radiosensitive) were established using clonogenic assays. ZD1839 was added for 24 hours prior to IR (0-10Gy). Data was analysed using unweighted non-linear least squares regression. Cell cycle kinetics and apoptosis were analysed using PI staining, Annexin-V FITC conjugates and flow cytometry.

**Results:** ZD1839 alone exhibited no growth inhibitory effect (p=0.9). The survival fraction at 2Gy ($SF_2$) was 0.88 and 0.66 for MGH-U1 and S40b respectively (p<0.001). With ZD1839 the $SF_2$ and $SF_{10}$ values were 0.83 and 0.012 for MGH-U1 and 0.42 and 0.0079 for S40b (p<0.001) consistent with a significant radiosensitising effect. No significant alteration in cell cycle kinetics was demonstrated. ZD1839 had a modest pro-apoptotic effect that was significantly enhanced in combination with IR.

**Conclusion:** ZD1839 enhanced anti-tumour potentiation of IR in bladder cancer cells. This was associated with a significant induction of apoptosis. This radiosensitising effect may have promising clinical implications for future treatment.

 © 2003 Cancer Research UK

### P151

A PHASE I TRIAL OF INTRA-VESICAL GEMCITABINE IN THE TREATMENT OF RECURRENT SUPERFICIAL TRANSITIONAL CELL CARCINOMA OF THE BLADDER.

Satish B. Maddineni*[1], Vijay Sangar[1], Christopher D.Betts[1],Kieran J. O'Flynn[1] & Noel W. Clarke[1,2].
[1]Salford Royal Hospitals NHS Trust, Salford, [2]The Christie Hospital NHS Trust, Manchester, UK.

**Introduction:** Superficial transitional cell carcinoma (TCC) accounts for 70-80% of all bladder cancer and has a high risk of recurrence and histological progression. Post-resection chemotherapy reduces recurrence by up to 50%. Systemic Gemcitabine is an active agent in bladder cancer. The aim of this study is to determine the maximum tolerated dose (MTD) and dwell time of intra-vesical Gemcitabine in patients with recurrent superficial TCC within 24 hours of trans-urethral resection.

**Methods:** Patients with recurrent superficial TCC of the bladder (G1-3pTa or G1-2pT1) received intra-vesical Gemcitabine instillations at a dose of 5mg/ml in 100ml NaCl with dwell times of 1 and 2 hours. Each dose level was studied in cohorts of 3 patients. Dose was escalated in 500mg increments. Patients underwent cystoscopy under general anaesthetic at 3 months.

**Results:** 16 patients have been studied with a mean age of 75 years. The 5mg/ml dose level was tolerated for both dwell times with no significant toxicity. 4 patients were studied at the 10mg/ml dose level but the maximum tolerated time to micturition was 40 minutes. Volume for dilution was reduced to 50ml of NaCl. 3 patients have been treated with a 1000mg in 50 ml of NaCl (20mg/ml) at both dwell times with no adverse effects.

**Conclusion:** Intra-vesical Gemcitabine is tolerated at 1000mg in 50ml of NaCl post-resection. Further studies are planned to assess this regional treatment as an adjuvant therapeutic option in the treatment of superficial carcinoma of the bladder.

### P152

A TARGETED RADIOTHERAPY/GENE THERAPY STRATEGY FOR BLADDER CANCER

Natasha Fullerton, Marie Boyd, *Lesley Wilson, Nicol Keith, David Kirk and Robert Mairs
Dept. of Radiation Oncology, Cancer Research UK Beatson Laboratories, Glasgow.

Targeted radiotherapy is the selective irradiation of tumour cells byradionuclides conjugated to tumour-seeking molecules. One such molecule, radiolabelled MIBG, is actively taken up via the noradrenaline transporter (NAT) only by tumours of neuroectodermal origin. Our aim was to apply this targeted radiotherapy to bladder cancer by gene transfer and limit NAT gene expression to bladder cancer cells by using a tumour specific telomerase promoter

The human bladder cancer cells, EJ138, were transfected with the NAT gene under control of the CMV, hTERT and hTERC promoters, exhibited a 11-, 34- and 36-fold enhancement, of active uptake of [$^{131}$I]MIBG. Administration of [$^{131}$I]MIBG resulted in dose dependant cell kill of EJ138 cells transfected with the NAT under control of the telomerase and CMV promoters. Biodistribution studies 48hours post injection of [$^{131}$I]MIBG into mice bearing tumour xenografts showed significantly increased uptake of [$^{131}$I]MIBG in the tumours derived from EJ138 expressing NAT under the control of telomerase promoter compared to negligible uptake in parental EJ138 xenografts.

These results show promise for a targeted radiotherapy / gene therapy approach for the treatment of bladder cancer, which would decrease side effects in the form of radiation toxicity from external beam irradiation and increase radiation dose to target organs, hence improving therapeutic efficacy.

### P153

A PHASE I/II STUDY OF GEMCITABINE AND CISPLATIN IN ADVANCED, METASTATIC, OR RELAPSED TCC OF THE BLADDER IN AN OUTPATIENT SETTING.

Syed A. Hussain*,[1,2] Deborah Stocken,[1] Peter Riley,[2] Ian Geh,[2] David Peake[2], David Spooner[2], Nicholas D. James,[1,2] Cancer Research UK Institute For Cancer Studies;[1] Queen Elizabeth Hospital,[2] Birmingham, United Kingdom.

**Introduction:** A randomized phase III trial of MVAC vs. gemcitabine/cisplatin (GC) (G 1,000 mg/m$^2$ d1,8,15 plus C 70 mg/m$^2$ d2, q 4 wks) indicated GC had similar efficacy and lower toxicity (*JCO* 2000). Significant hematologic toxicities in GC arm were on d15, causing dose adjustments in 37% of cycles. We are conducting a phase I/II dose escalation trial with GC on a 21-day cycle, with G and C split between d1,8. **Objectives:** To define the MTD and toxicity; determine response, and progression-free and overall survival. **Methods**: 32 pts with locally advanced, relapsed, or metastatic disease received: dose level 1: G/C 1000/35; level 2: 1100/35; level 3: 1200/35; level 4: 1200/45 mg/m$^2$ (G and C given on d1,8 every 3 wks). Eligibility: ECOG PS 0-2, adequate bone marrow/liver function, GFR > 40 mL/min. **Results:** 32 pts enrolled; 28 evaluable. Characteristics: M/F 22/10, median age 66 yrs (range 41-79), 16/32 GFR < 60 mL/min. Stage: N+ (22), M1 (19). Prior therapy: cystectomy (7); RT (8); progression after platinum-based chemo (3). DLT was hematologic (grade 4 thrombocytopenia) at level 2; study continued at level 1. Hematologic toxicity: level 2 (9 pts): grade 4 neutropenia (2) and thrombocytopenia (3) were DLTs. Level 1 (23 pts): grade 3 anemia (4) and neutropenia (9), and grade 3/4 thrombocytopenia (10/2). Nonhematologic toxicity: grade 1 perforation (1), grade 4 GI bleed (1) and acute abdomen (1), grade 3 nausea (2). Of 125 cycles, platelets were <100 (d15) in 53; neutrophils <0.5, platelets <50 in 23. Only 3 cycles deferred due to grade 4 neutropenia; 2 for renal toxicity (chemo instituted post hydration). 3 deaths occurred: 1 GI perforation (steroid antiemetic); 1 GI bleed (warfarin for DVT secondary to massive pelvic lymphadenopathy, ultimately found to have pyloric ulcer); 1 pt developed acute abdominal pain, and died after exploratory surgery. 1 pt was noncompliant. Overall ITT response was 68% (79% [19/24] for assessable pts), with 4 CRs (14%) and 15 PRs (54%). 23 pts are alive, median follow-up 6.0 mos (range 1.0-18.1). Survival analysis will be presented (12-mo minimum follow-up). **Conclusion:** G plus C every 3 wks is active and well tolerated in an outpatient setting, even in pts receiving prior platinum-based regimens and with poor renal reserve.

### P154

LARGE CELL NEUROENDOCRINE LUNG CANCER: A DIFFERENT CLINICAL ENTITY?

Marianne Nicolson and Keith Kerr on behalf of the Aberdeen Lung Cancer Group, ANCHOR Unit, Aberdeen Royal Infirmary, Aberdeen AB25 2ZN

Introduction: Large cell neuroendocrine carcinoma of the lung has recently been redefined by the World Health Organisation. These tumours share morphological characteristics with large cell carcinomas and immunohistochemical features with neuroendocrine tumours. Clinically they behave more like small cell lung cancers and may require a different therapeutic approach. We present the data from 19 such patients (pts) diagnosed and treated between 1996 and 2002 in Aberdeen Royal Infirmary.

Method: Pathology listings were obtained for pts with large cell neuroendocrine cancers. Notes review was undertaken to confirm that these were primary lung cancers. Data on treatment and survival are presented.

Results: There were 12 males and 7 females. Age range was 42 to 75 years. Stage at presentation was I/II 7 pts, IIIA 3 pts, IIIB 3 pts and IV 6 pts. Treatment was surgery in 10 (1 segmental resection, 5 lobectomy and 4 pneumonectomy), primary chemotherapy in 6, primary radiotherapy in 2 and 1 pt refused treatment. For the surgical pts, 2 of the 4 who had pneumonectomy are alive at 2 and 15 months out; two died at 14 and 15 months from disease relapse. Of the lobectomy pts, three relapsed at 9, 13 and 18 months; follow-up data are incomplete on the other two and the one pt who had segmental resection. Of the patients with stage IIB and IV disease, one pt achieved CR maintained at 4 months, two had PR and three stable disease. The updated survival of these patients and those treated with primary radiotherapy will be presented with a review of their pathology characteristics.

© 2003 Cancer Research UK

### P155

STROMELYSINS ARE DIFFERENTIALLY OVEREXPRESSED IN HUMAN LUNG CANCERS: POTENTIAL FOR THERAPEUTIC INTERVENTION J M Seargent[1], P M Loadman[1], S W Martin[1], S Tijani[2], J H Gill[*1]. [1]Tom Connors Cancer Research Centre, University of Bradford, UK; [2]Department of Histopathology, Bradford Royal Infirmary, Bradford, UK

The degradation of the extracellular matrix (ECM) is central to tumour growth, invasion and metastasis. One class of enzymes responsible for such activities are the matrix metalloproteinases (MMPs), which include stromelysins 1 and 2 (MMP-3, -10). Knowledge of stromelysin 1 and 2 (ST-1 and –2) expression in human lung carcinomas and their relationship to tumour characteristics is limited. Such information is likely to have both prognostic and diagnostic implications for human lung cancer. Understanding the expression profile of the stromelysins and their proposed tumour specific activity also has implications for the development of selective anticancer drug development. This study analysed ST-1 and –2 expression in human lung tumours of various grade, stage and type, initially to determine any relationship between expression and tumour characteristics and secondly to establish their potential as therapeutic targets. ST-1 and –2 protein expression was studied immunohistochemically using antibodies specific for either ST-1 or ST-2. Formalin-fixed lung tumours from fifty patients representing all grades, stage, type and metastatic potential were studied. Consent to use these specimens in the current study was obtained from the relevant Local Research Ethics Committee. Differential tumour specific expression of both ST-1 and ST-2 were observed in all cases. Expression of ST-2 was predominantly in the tumour epithelia whereas ST-1 was located primarily within the tumour stromal compartment. No correlation was observed between expression level and tumour grade or stage in either case. These data demonstrate that stromelysins 1 and 2 are expressed in different tumour regions, demonstrate tumour specific expression and are valid targets for anticancer drug development.

### P156

REDUCTION IN TESTICULAR GERM CELL CANCER SIZE WITH INCREASING AWARENESS.

Jeetesh M Bhardwa*, RTD Oliver, Vinod Nargund, D M Berney

Improved overall survival of germ cell cancer was observed to be associated with increasingly early diagnosis and reduced tumour size[1]. Over the last 6 years there has been considerable increase in public awareness of testis cancer. This abstract reviews trends in testis tumours size to see if there has been an impact of the increased awareness on tumour size.

Material & Methods: Pathology Department reports measurements in patients undergoing orchidectomy were reviewed and correlated with year of diagnosis.

Results: The median diameter of tumour size has reduced from 24.5mm in 32 patients treated 1996 –98, to 19.5mm in 37 patients treated 1999 to 2002.

Conclusion: These data confirm continuation of the trend previously observed when median fell from 48mm in 1978 –83, to 45mm in 1984-85, 44mm in 1989-94[1] suggesting that improved health education in the susceptible male population had resulted in a reduction in testicular tumour size and this has implications for management of testicular tumour, such as use of testis conserving surgery and chemotherapy in patients with reduced germ cell function at presentation.

**Reference**: Testis conservation studies in germ cell cancer justified by improved primary chemotherapy response and reduced delay, Oliver RT. Ong J. Blandy JP. Altman DG 1978-1994. [Journal Article] British Journal of Urology. 78(1): 119-24, 1996 Jul.

### P157

TREATMENT OPTION FOR MALIGNANT HEMANGIO-ENDOTHELIOMAS OF THE THYROID.

Walter Rhomberg*, Franz Boehler, Helmut Eiter, Heinz Fritzsche, Gerhard Breitfellner. Correspondence: W.R., Department of Radio-oncology, General Hospital, A-6800 Feldkirch, Austria

**Background:** Malignant hemangioendothelioma [MHE] of the thyroid is a rare tumour predominantly seen in areas with endemic goitre such as the Alpine regions. The tumour is regarded as radio-resistant and its prognosis is reported to be dismal (MST 2-4 months) [Ladurner D et al (1990) Wien Klin Wochenschr 102:256].

**Patients and methods:** Between 1982 and 1999, 12 cases with immunohistochemically confirmed MHE of the thyroid were referred for postoperative or palliative radiotherapy. By surgery, clear margins had been achieved in 5, microscopic residues were left in 3, and gross residual disease in 3 patients. One patient had an inoperable primary tumour. Postoperative radiotherapy between 54 and 65 Gy was given to 8 cases, 6 of them received razoxane (125 mg twice daily by mouth on radiation days). Razoxane is a radio-sensitizer and an agent which can normalize tumour blood vessels.

**Results:** Local tumour control was achieved in 11 of 12 patients; 5 of 12 lived longer than 5 years. The median survival time of all cases is 14 months. If 3 cases with a primary metastasis were left out from the analysis, the median survival is 70 months. The tolerance to the combined treatment was good to fair.

Fibrinogen, factor VIII and factor VIII-antigen may eventually serve as „markers“ during the follow up. It may also be of interest that 5 of 12 patients were exposed to vinyl chloride and other poly-meric materials during their occupational life.

**Conclusions:** The outcome of this disease may not be uniformly grim, and the resistance to radiotherapy reported in the literature may be questioned. Razoxane may have contributed to the high local control rate and thus perhaps to an alteration of the natural history of the disease to some extent.

### P158

A SMALL PROSPECTIVE STUDY OF CHORDOMAS TREATED WITH RADIOTHERAPY AND RAZOXANE.

Walter Rhomberg*, Franz Boehler, Hansjörg Novak, Susanne Dertinger, Gerhard Breitfellner. *Dept. of Radiooncology, General Hospital, Carinagasse 47, A-6800 Feldkirch / Austria

Purpose: To evaluate the local effect of conventional photon irradiation in chordomas if the radiosensitizing agent razoxane is added. The rationale for this procedure were low local control rates by photons (27%, textbooks), and improved responses previously seen in soft tissue and chondrosarcomas with photons + razoxane.

Materials and methods: Between 1988 and 1996 five patients with histologically confirmed chordomas of the base of the skull or the spine (3 females, 2 males) were irradiated with high energy photons together with razoxane. The median total radiation dose was 63 Gy (range 54-67) with single fractions of 2 Gy. Razoxane was concurrently given at a dose of 125 mg twice daily by mouth on radiation days; the median total dose was 7.6 g per patient. The drug was started 3-5 days before the first irradiation.

Results: After a potential median follow up time of 10 years, 3 of the 5 patients are alive and show neither symptoms nor signs of a recurrence in CT or MR images. One patient died after 8 years with a persistent sacral chordoma from cardiac insufficiency, and another patient died after 6.5 years from a haemorrhage after surgery for recurrence. All patients remained locally controlled for 5 years and longer(5, 5.5+, 6.4, 11+ and 13+ years, respectively). Objective tumour regressions were noted in 3 of 4 patients with measurable disease. – Acute side effects were mucosal reactions, 2 of 5 patients developed a leukopenia of grade 3 WHO due to razoxane. There were no serious long term complications.

Conclusions: Although the patient series is small, there is an interesting trend in local control and survival. The cases are unselected, and the follow-up time is of considerable duration. The treatment is easy to perform at any institution.

© 2003 Cancer Research UK

### P159

STREPTOZOCIN, 5-FLUOROURACIL/FOLINIC ACID AND CISPLATIN (FCiSt) FOR ADVANCED NEUROENDOCRINE TUMOURS

Debashis Sarker*[1], Martin Williams[2], John Buscombe[3], Martyn Caplin [4], Daniel Hochhauser[1], Richard Begent[1], Tim Meyer[1] Depts. of Oncology[1], Radiology[2], Nuclear Medicine[3] and Gastroenterology[4], Royal Free Hospital(RFH), London, NW3 2QG

**Background:** Streptozocin-based combination chemotherapy has become standard therapy for advanced neuroendocrine tumours (NET), particularly rapidly proliferating pancreatic NET. Cisplatin also has proven efficacy, in particular for poorly differentiated NET. At the RFH we have used a novel combination of these agents with 5-Fluorouracil (5FU) and Folinic Acid (FA). **Patients and Methods:** A retrospective review of 25 patients with advanced NET (14 pancreatic NET, 5 metastatic NET of unknown primary, 6 metastatic carcinoid), from 1996-2003. Sites of metastatic disease were liver (14), locoregional lymphadenopathy(10), peritoneum(2), lung(2) and bone(1).The regimen was FA 45mg, 5-FU 500mg/m² iv bolus, Cisplatin 70mg/m², Streptozocin 1000mg/m², q21 days. Mean age at diagnosis was 55.7 years (range 26-77 yrs), median number of cycles administered 6 (range 1-12). Response was assessed by RECIST, and toxicity by CTC criteria. **Results:** 23 of 25 patients had assessable disease.8 patients (34%) had a partial response, 9 (39%) stable disease, and 6 (26%) progressive disease. 11 patients had grade3/4 toxicities: diarrhoea (3), vomiting(3), neutropaenia (2), neuropathy(1),fatigue(1), thrombocytopaenia (1). There was 1 episode of neutropaenic sepsis and no treatment related deaths. Median time to progression was 12 months; median overall survival has not been reached. **Conclusions:** In this heterogeneous group of patients, FCiSt shows comparable response rates to previous regimens with acceptable toxicity. Phase III trials are required to further compare FCiSt with other regimens for NET.

### P160

THE ROLE OF SPLENECTOMY IN LYMPHOMA – A SINGLE INSTITUTION EXPERIENCE.

Simon J Crabb*, Tim M Illidge, Peter WM Johnson.
Oncology Unit, Southampton University Hospital NHS Trust.

Background: The indications for splenectomy in lymphoma are poorly defined. We have therefore reviewed our practice to assess the possible role.

Methods: We performed a retrospective review on all lymphoma patients from our institution who had splenectomy since 1990.

Results: Twenty nine patients were identified as shown below.

| Diagnosis | n | Splenectomy Indication | n |
|---|---|---|---|
| Small lymphocytic lymphoma | 13 | Diagnosis/sole site | 9 |
| Marginal zone lymphoma | 6 | Myelosuppression | 8 |
| Follicular lymphoma | 4 | Hypersplenism | 7 |
| Mantle cell lymphoma | 2 | Sole relapse site | 2 |
| Diffuse large B cell lymphoma | 1 | Unclear | 3 |
| Peripheral T cell lymphoma | 1 | | |
| MALT lymphoma | 1 | | |
| Hodgkins disease | 1 | | |

Mean age was 60 at diagnosis. Nine patients had transient peri-operative complications (5 infective, 2 cardiovascular, 1 urinary retention) with no deaths. Median survival is 59 months (8–178) from diagnosis, and 42 (6–109) post-splenectomy. 12 had splenectomy as primary therapy (2 with chemotherapy) with 7 (58%) in complete remission at a mean follow up of 68 months (36–101). 7 had splenectomy as second line therapy with 3 free from progression (9, 19 and 24 months follow up) and 4 relapsed (at 1, 2, 21 and 42 months). 10 patients have had no therapy post-splenectomy at mean follow up of 56 months (9–109). 5 patients were transfusion dependant which resolved in each post-splenectomy. Platelet mean pre-splenectomy was $90x10^9$/L (11-217, six <50) and 424 (43–1320, one<50) one month post.

Conclusion: Splenectomy has a role in lymphoma therapy when the spleen represents the dominant disease site. It may be curative or provide durable relapse free survival, and has tolerable sequelae.

### P161

NON-HODGKIN'S LYMPHOMA OF THE OCULAR ADNEXAE; THREE DECADES OF EXPERIENCE AT A SINGLE UK CENTRE.

Anne C Armstrong*, John Chapman, Shankar S. Banerjee, Joan L Noble, Brian Leatherbarrow, W David J Ryder, Richard Bonshek, David P Deakin, Richard A Cowan and John A Radford.
CRUK Dept of Medical Oncology, Depts of Histopathology and Clinical Oncology, Christie Hospital, Manchester UK.

Between 10/77 and 12/00, 106 pts with non-Hodgkin's lymphoma (NHL) involving the ocular adnexae were subjected to histology review, staged and treated. Clinical data for the entire cohort was obtained and, where possible, biopsies were reviewed a second time and categorised according to the WHO classification.

A second histological review was possible for 55 of the 106 cases. Confirmed histological subtypes were follicular (n=7), diffuse large B-cell (n=6), lymphoplasmacytic (n=5) and mantle cell (n=3). The remaining 34 cases were B-cell lymphomas of indolent type that were thought likely to be extra-nodal marginal zone (MZL) but could not be confirmed as such. Clinical data for all 106 pts were examined. The median age at presentation was 66 yrs (range 11-92) and 58 were female and 48 male. 77 pts had stage IE disease, 14 stage II, 1 stage III and 14 stage IV. Treatment was radiotherapy (RT) alone in 81 cases, chemotherapy (CT) alone in 9 cases and RT and CT in 12 cases. With a median follow-up for survivors of 6.5 yrs (range 1.3-21.4), 62 pts are alive and 44 have died (18 from NHL, 6 from other cancers and 20 from non-malignant causes). Median overall survival (OS) for the whole group is 11 yrs (95% CI 8.1-17.8); 5yr OS for the 91 pts with stages I/II is 78% and 54% for the 15 pts with stages III/IV.

The majority of ocular adnexal lymphomas are of indolent B-cell type, stage IE at diagnosis and have a good prognosis following treatment with RT. There is a high incidence of probable MZL and the molecular genetics of this subgroup are being investigated.

### P162

THE EFFECT OF PROTEASOME INHIBITION WITH BORTEZOMIB(VELCADE™) IN B CELL LYMPHOMA CELL LINES

SJ Strauss*[1], W Liu[1], L Maharaj[1], R Shringarpure[2], C Anderson[2], D Schenkein[3] TA Lister[1], SP Joel. Dept. Medical Oncology, St Bartholomew's Hospital, West Smithfield, London[1]; Jerome Lipper Multiple Myeloma Center, Dept Medical Oncology, Dana-Farber Cancer Institute, Boston, MA, USA[2], Millennium Pharmaceuticals, Boston, MA, USA[3].

**Introduction**: The proteasome degrades ubiquitinated proteins involved in regulating cell growth, the cell cycle and apoptosis. Bortezomib (Velcade™, formerly known as PS-341) is a proteasome inhibitor with cytotoxic activity in a number of malignant cell lines, and in a phase III trial of patients with multiple myeloma. The effect of bortezomib in 3 lymphoma cell lines was investigated, to examine efficacy, and investigate factors influencing activity. **Methods and Results:** The effect of bortezomib on proteasome activity was examined by chymotryptic enzyme analysis. Proteasome inhibition was apparent at 2 hrs and comparable in all cell lines: DHL-4: 86 ± 3%, DHL-6: 78 ± 9%, and DHL-7: 84 ± 1%. Bortezomib induced concentration-dependent growth inhibition and reduction of cell viability in all cell lines. The IC50 for % viability after a 3-day drug exposure was: DHL-4: 26nM, DHL-6: 3nM and DHL-7: 7nM. Cell cycle analysis by flow cytometry revealed a concentration-dependent increase in apoptosis (A) in the more sensitive DHL-6 and DHL-7 cells with a reduction in G1 and G2. In contrast, similar effective doses resulted in a G2 block in DHL-4 cells with a smaller increase in apoptosis (Table).

**Table: Effect of bortezomib on the cell cycle in DHL-4 and DHL-7 cells**

| | DHL-4 | | | DHL-7 | | |
|---|---|---|---|---|---|---|
| | 0nM | 25nM IC50 | 100nM 4xIC50 | 0nM | 10nM IC50 | 30nM 4xIC50 |
| **A** | 2 ± 1 | 10 ± 3 | 17 ± 4 | 5 ± 2 | 17 ± 4 | 41 ± 1 |
| **G1** | 57 ± 5 | 52 ± 1 | 44 ± 1 | 56 ± 3 | 45 ± 8 | 30 ± 2 |
| **S** | 18 ± 1 | 4 ± 1 | 8 ± 1 | 19 ± 1 | 20 ± 1 | 14 ± 2 |
| **G2** | 21 ± 2 | 34 ± 1 | 32 ± 3 | 18 ± 1 | 13 ± 1 | 13 ± 1 |

**Conclusion:** Bortezomib is effective at low nanomolar concentrations in 3 lymphoma cell lines. Although proteasome inhibition was equivalent, sensitivity varied and resulted in different effects on the cell cycle. Possible mechanisms for this difference, including the role of p53 and effects on cell cycle regulatory proteins are being examined.

**P163**

HIGH DOSE INTERFERON-α-2b ADJUVANT TREATMENT FOR MELANOMA: THE UK EXPERIENCE

*Effie Katerinaki, Bihani Kularatne, Rachel Hambly, Lesley Turner, Barry W. Hancock and Paul Lorigan on behalf of the UK collaborators
Academic Department of Clinical Oncology, Weston Park Hospital, Sheffield, S10 2SJ, UK

The objective of this study was to review the toxicity and feasibility of high dose Interferon-α-2b adjuvant treatment for melanoma patients reported to a national database from 18 UK centres.

One hundred and twenty three patients (median age 47 years) were reported for the one month i.v. induction phase. Fever, fatigue, leucopenia and hepatotoxicity were the commonest toxicities. Dose delays were required for 33% and dose reductions for 45% of the patients at least once and the commonest causes were neutropenia and hepatotoxicity. The majority of the patients (90%) completed the 4-week regimen.

Sixty nine patients (median age 42 years) were reported for the eleven month maintenance phase. No data for dose modifications were available. Grade I and II constitutional symptoms, leucopenia and hepatotoxicity were mostly reported and the incidence declined during the course of treatment. 68% of the patients who entered this phase completed treatment on protocol until one year or disease progression/death.

In conclusion, high dose Interferon-α-2b treatment was feasible but the associated toxicity was significant and similar to that previously reported by the ECOG studies. This regimen is the standard of care for stage IIB/III melanoma in USA and is gaining acceptance in UK. Close treatment monitoring is essential, and the toxicity must be carefully balanced against benefit to patients.

**P164**

TNF-α INCREASES HUMAN MELANOMA CELL INVASION AND MIGRATION *in vitro* -THE ROLE OF PROTEOLYTIC ENZYMES

*Effie Katerinaki, Gareth Evans, Paul Lorigan and Sheila MacNeil. Academic Department of Clinical Oncology, Weston Park Hospital, Sheffield, S10 2SJ.

**Background:** We have previously shown that the proinflammatory cytokine TNF-α upregulates human melanoma cell attachment to ECM substrates, invasion through fibronectin and integrin subunit expression (Zhu *et al*, *J Invest Dermatol* 2002; 119: 1165-1171).

**Aim:** The aim of this study was to examine whether the effects of TNF-α on melanoma cell invasion and migration *in vitro* are mediated by upregulation of, or activation of degradative enzymes.

**Materials and methods:** We used human recombinant TNF-α to study its effect on the invasion of the human cutaneous melanoma cell line HBL through fibronectin over 20 hours, cell migration over 24 hours ("scratch wound" assay), expression/activation of MMPs-2 and MMP-9 (detected by gelatin zymography) and general protease activity (detected by quenched fluorescent substrate assay) measured at 24 hours. We also used the general protease inhibitor $\alpha_2$ macroglobulin to study its effect on HBL cell invasion and migration.

**Results:** TNF-α significantly increased melanoma cell invasion through fibronectin by +35% and cell migration on plastic by +21% above control (non-treated cells). The cells expressed low levels of latent MMP-2 (and no MMP-9) and general proteolytic enzymes that were not activated or upregulated by TNF-α. However, the TNF-α stimulated invasion through fibronectin and migration were inhibited by $\alpha_2$ macroglobulin.

**Conclusions:** TNF-α significantly increases melanoma cell invasion and migration *in vitro.* This effect may be partially mediated by upregulation of localised peri-cellular proteolytic enzyme activity not readily detected in general biochemical assays.

**P165**

THE EFFECT OF RECOMBINANT ERYTHROPOEITIN ON RENAL CANCER CELL LINES AND ITS ROLE IN CHEMOSENSITIVITY.

Thomas Powles, Wai Liu, Jonathan Shamash, Tim Oliver and Simon Joel. New Drug Study Group, St Bartholomew's Hospital, London, UK.

Recombinant erythropoetin (rEpo) has an important role in the management of cancer patients. However, the effects at a cellular level are still poorly understood. We examined the effect of rEpo alone, and in combination with cytotoxic chemotherapy, in seven cell lines, including 3 renal lines. In particular, we assessed the relationship between rEpo receptor expression and activation of biochemical pathways regulating cell kill; specifically Bcl 2, which can be up regulated by other growth factors resulting in chemoresistance. Our results showed that rEpo did not significantly alter cell viability, cell cycle dynamics or the rate of cell proliferation. Flow cytometric analyses showed differences in the level of receptor expression (range: 3.4 ± 0.7 to 39.5 ± 2.2), with the renal cell lines having the highest rEpo receptor level (>17.5 ± 5.5). As there were no changes in the cell parameters, we investigated the activation of Erk by means of immunoblot analyses. Phosphorylated Erk was up regulated following culture with rEpo in the cell lines expressing a high level of the receptor. However there was no subsequent up regulation of Bcl-2 protein levels. The culture of cells pre-treated with rEpo with cytotoxic EC50 concentrations of cisplatin did not result in an altered magnitude of % cell kill. These results highlight the complexity of the rEpo-signalling pathway, and suggest that unlike other growth factors, Bcl-2 is not up regulated following its use. Therefore, the use of rEpo appears to be safe in this setting, even in renal tumours that express higher levels of the receptor.

**P166**

**WITHDRAWN**

© 2003 Cancer Research UK

**P167**

ESSENTIAL REQUIREMENT OF APOLIPOPROTEIN J (CLUSTERIN) SIGNALLING FOR IKB EXPRESSION AND MODULATION OF NF-KB ACTIVITY: IMPLICATIONS FOR NEUROBLASTOMA

Giorgia Santilli, Bruce J. Aronow and Arturo Sala*

Molecular Haematology and Cancer Biology Unit, Institute of Child Health, UCL, London WC1N 1EH, United Kingdom.
a.sala@ich.ucl.ac.uk

ApolipoproteinJ/clusterin is an enigmatic protein induced in inflammation, apoptosis and cancer. In spite of extensive studies, its biological function has remained controversial. We have recently observed that Apolipoprotein J is directly regulated in neuroblastoma cells by B-MYB, a protooncogenic transcription factor involved in cell proliferation and survival. Here we show that Apolipoprotein J can modulate survival and in vitro and in vivo invasive potential of neuroblastoma cells. Since it has been reported that these biological properties can be regulated by NF-kB, we explored the possibility that Apolipoprotein J might interfere with NF-kB signalling.

Remarkably, exogenous Apolipoprotein J expression strongly inhibited NF-kB activity in human neuroblastoma cells and murine embyonic fibroblasts by stabilising inhibitors of NF-kB (IKBs). Steady state levels of IKB proteins are drastically reduced in mouse embryo fibroblasts after disruption of the Apolipoprotein J gene. Absence of ApoJ caused reduced IKB expression, a TNF-dependent increase in NF-kB activity, and increased transcription of the NF-kB target gene c-IAP.

These results suggest that an unexpected physiological role of Apolipoprotein J is to suppress NF-kB signalling through stabilisation of IKBs.

**P168**

EFFECTS OF KETOCONAZOLE AND ACITRETIN ON RETINOID ISOMERISATION AND METABOLISM IN NEUROBLASTOMA

Jane L. Armstrong*, Gareth J. Veal, Christopher P. F. Redfern and Alan V. Boddy. Northern Institute for Cancer Research, University of Newcastle upon Tyne, Newcastle upon Tyne NE2 4HH.

Treatment with 13-cis retinoic acid (13cisRA) has been shown to significantly improve clinical outcome in high-risk neuroblastoma (nbl). Isomerisation to all-trans retinoic acid (ATRA) potentiates the cellular activity and growth inhibitory effects of 13cisRA in nbl cells *in vitro*. The major route of RA catabolism is thought to be oxidation mediated by specific P450 enzymes. Cellular retinoic acid binding proteins (CRABPs) are also thought to regulate free intracellular ATRA levels by promoting metabolism. We have evaluated the effect of acitretin, an analogue of RA known to bind strongly to CRABPs, and the P450 inhibitor ketoconazole, on 13cisRA isomerisation and metabolism in nbl cells *in vitro*.

SHSY5Y cells were incubated with either acitretin (100 μM) or ketoconazole (20 μM) for 24h prior to incubation with 13cisRA (10 μM) for a further 24 h. Cell pellets were collected and intracellular retinoids extracted prior to analysis by HPLC. CRABP expression was assessed by western blot analysis.

Pre-incubation with acitretin resulted in a selective increase in intracellular ATRA concentrations (26-fold). The extent of this increase correlated with induction of CRABP in the cells studied. Pre-incubation with ketoconazole lead to an increase in both intracellular ATRA (5-fold) and 13cisRA (2.5-fold) levels suggesting an inhibition of metabolism of both isoforms.

These data indicate that the extent of 13cisRA isomerisation and metabolism in nbl cells can be modulated by acitretin and ketoconazole.

**P169**

DIFFERENTIAL SIGNALING VIA SURFACE IgM IS ASSOCIATED WITH $V_H$ GENE MUTATION STATUS AND CD38 EXPRESSION IN CHRONIC LYMPHOCYTIC LEUKEMIA (CLL).

Stuart Lanham*, Terry Hamblin, David Oscier, Rachel Ibbotson, Freda Stevenson, and Graham Packham.

Somers Cancer Research Building, Southampton General Hospital, Tremona Road, Southampton, SO16 6YD, UK

Although $V_H$ gene mutation status and CD38 expression are important prognostic factors for CLL, the biological differences which determine variable disease course have not been identified. By measuring increases in Syk and global tyrosine phosphorylation in cells treated with anti-IgM, we show that the ability to signal through the B-cell receptor significantly correlates with $V_H$ gene status (p=0.003) and CD38 expression level (p=0.00009). Overall, 71% of unmutated cases (n=21) responded compared to 30% of mutated cases. Failure to signal through ligation of IgM could be circumvented by ligation of IgD (19/29 samples tested) or the BCR-associated molecule CD79a (23/29 samples tested). Retention of responsiveness to IgM-ligation may contribute to the poor prognosis of the unmutated and CD38 positive subsets. Our results also suggest that multiple mechanisms underlie non-responsiveness to IgM-ligation in CLL. The pattern of differential signaling indicates possible differences in the molecular organization of the BCR, and points to features of anergy in the mutated subset.

The prognostic power of the in vitro response to IgM ligation remains to be determined in a large series, but the simple technology involved may present an alternative/additional test to $V_H$ gene analysis for predicting clinical course.

**P170**

DEVELOPMENT OF A TOUCH SCREEN QUESTIONNAIRE APPROACH FOR MEASUREMENT OF PAIN ASSESSMENT SCALES IN CANCER PATIENTS WITH BONE METASTASIS

Iain M Goodhart, Janet E Brown*, Susan Ellis, Sandra Gutcher, O Prakash Purohit, Robert E Coleman, Academic Unit of Clinical Oncology, Cancer Research Centre, Weston Park Hospital, Sheffield, UK.

Pain is one of the most common features in patients with cancer and has a significant impact on quality of life, but it is often poorly assessed. For patients with metastatic bone disease of the breast and prostate, survival is often measured in years rather than months and the assessment of bone pain and its reduction are major clinical goals.The success of treatment in such patients is often assessed by measuring the change in objective pain scores, which take the form of paper questionnaires and pain scales, which can be time consuming to complete and score correctly. We report a pilot study to develop and evaluate a patient-operated, computerised, touch-screen, pain questionnaire which was aimed at improving the efficiency of data entry and the quality of the patient/questionnaire interface.

Touch screen questionnaires for this application were not commercially available and the script enabling the questionnaire to operate was specifically written for the study. Pain scores from the touch screen approach were compared with those from two existing paper format pain questionnaires, the Wisconsin Brief Pain Inventory and a locally developed pain scale. With Ethical Committee approval each patient had all three pain scores measured at four separate visits.

Statistical analyses of the data were conducted on completed data sets from 29 patients with bone metastases from breast or prostate cancer. 86% of patients were happy to use the touch screen format, with only 14% of patients preferring the paper questionnaires. Moreover, the touch screen version could be completed in around half the time of the paper version, with fewer unanswered questions. In addition, the touch screen version automatically computed the pain scores. It was shown that touch-screen and paper linear analogue scales were comparable with a mean score difference of 5.5% and Pearson product moment correlation coefficients in the order of 0.9. It is envisaged that this work will lead to the further development of refined touch-screen questionnaires for assessment of pain scores.

© 2003 Cancer Research UK

**P171**

LEVOMEPROMAZINE VERSUS DEXAMETHASONE WITH METOCLOPRAMIDE IN THE PREVENTION OF CISPLATIN INDUCED DELAYED EMESIS. PRELIMINARY RESULTS OF A RANDOMIZED STUDY.

Joseph Sgouros*, Helen Neville-Webb, Suzan Bunton, Mike J. Lind, Anthony Maraveyas, Academic Department of Oncology, Princess Royal Hospital, Hull, UK

We have previously reported that Levomepromazine is a useful agent for chemotherapy induced delayed vomiting salvage. The primary endpoint of this study is to compare Levomepromazine (Nozinan®) at its oral novel formulation of 12.5mg bd for 3 days (Arm A) with the standard combination of Dexamethasone 8mg bd for 2 days followed by 4mg bd for 2 more days and Metoclopramide 10mg tds for 5 days (Arm B) in the prevention of ECF/ MCF induced delayed nausea and vomiting. Eligible patients have gastric/oesophageal carcinoma with no GI compromise and no pre-treatment vomiting. Study antiemetics are commenced 16 hours post Cisplatin. NCI common toxicity criteria are used and patients with ≥grade 2 N&V are being crossed over to the alternative arm Since no phase II data existed, an early analysis when 40 patients had been recruited was deemed necessary. So far 15 patients (80% men, mean age 59, median PS 80%) have been allocated in Arm A and 17 (94% men, mean age 66, median PS 80%) in Arm B. Complete control of delayed nausea and vomiting in the 1$^{st}$ cycle of chemotherapy was achieved in 54% and 69% of patients in Arm A and 26% and 40% in arm B respectively (NS). In subsequent cycles the complete control of nausea and vomiting in both arms was almost the same. 30% of patients in Arm A and 20% in Arm B needed to be crossed over after the 1$^{st}$ cycle. More patients in Arm B had insomnia (10% vs 47%) ($p<0.005$). Recruitment of patients continues.

**P172**

PROTEOLYTIC DIGESTION OF A TUMOUR-DERIVED LIPID MOBILISING FACTOR AND SUBSEQUENT IDENTIFICATION OF AN ACTIVE FRAGMENT.

Paul M SANDERS* and Michael J TISDALE; Pharmaceutical Sciences Research Institute, Aston University, Birmingham, UK B4 7ET

Cancer cachexia comprises unintentional and debilitating weight loss that accompanies specific tumour types. Cachexia reduces efficacy of chemotherapy, prognosis and patient quality of life.

Adipose depletion in cachexia is mediated by a 43KDa glycoprotein Lipid Mobilising Factor (LMF). LMF appears homologous to the endogenous peptide Zinc Alpha-2 Glycoprotein (ZAG).

To unlock the therapeutic potential of this molecule and to enable adequate receptor-interaction studies, it was investigated whether the molecule could be fragmented whilst retaining lipolytic activity.

LMF was purified from the urine of pancreatic cancer patients and loaded onto an immobilised trypsin HPLC column. The eluate was subjected to sephadex G50 size exclusion chromatography. Resulting fractions were assayed for lipolytic activity by monitoring glycerol release from isolated murine epidydimal white adipose tissue.

A low molecular weight fraction was produced which retained lipolytic activity. Active fragment size was estimated at approximately 6-12 KDa following G50 column standardisation and fraction electrophoresis against ultra low molecular weight markers. A digestion of ZAG in this manner also yielded a similar active fragment, providing evidence for homology between LMF and ZAG.

**P173**

ACTIVATION OF PKC AND NFkB BY A PROTEOLYSIS INDUCING FACTOR (PIF) IN CANCER CACHEXIA.

*StaceyM. Wyke, Helen J. Smith and Micheal J. Tisdale.Aston University, Aston triangle, Birmingham, B4 7ET.

Proteolysis Inducing Factor (PIF) is a 24kDa glycoprotein that can be isolated from cachexia-inducing murine and human tumours and from the urine of cachectic cancer patients. PIF causes breakdown of skeletal muscle, both *in vivo* and in vitro through the activation of the ubiquitin – dependant proteolytic pathway. The mechanism by which this occurs was studied in C2C12 myotubes as a surrogate model for skeletal muscle. PIF increased 26S proteasome activity (represented by 'chymotrypsin like' activity in C2C12 myotubes at concentrations between 1 – 20nM and maximum stimulation was achieved at 4.2nM (p=<0.001). This effect was attenuated by the PKC inhibitors Ro31-8220 and calphostin C.

The PKC inhibitor calphostin C demonstrated inhibition of IkBa degredation in response to PIF, inhibiting translocation of NFkB to the nucleus. Calphostin C also inhibited translocation of PKC in response to PIF. The action of PIF was also attenuated by the NFkB inhibitor curcumin, with regard to chymotrypsin like activity, 20S and E2 expression, suggesting that NFkB may be involved in the regulation of the ubiquitin – dependant proteolytic pathway.

These results suggest that the PKC and NFkB pathways are exploited by PIF in cancer cachexia.

**P174**

THE VICTOR TRIAL (VIOXX® IN COLORECTAL CANCER THERAPY, DEFINITION OF OPTIMALREGIME)

*Stuart Pendlebury, Francesca Duchesne, Katharine A Reed, Justine L Smith, David J Kerr (On behalf of the VICTOR Trial Advisory Group)

Epidemiological, clinical, animal and laboratory studies have demonstrated that non-steroidal anti-inflammatory drugs (NSAIDs) may prevent colorectal carcinogenesis. However the use of traditional NSAIDs, which inhibit both forms of cyclo-oxygenase (COX-1 and COX-2) has been restricted because of their gastrointestinal toxicity. The recently developed COX-2 selective NSAIDs exhibit a reduced incidence of gastrointestinal side effects when compared to traditional NSAIDs whilst retaining their anti-neoplastic properties, raising the possibility that they may be effective not only for chemoprevention but also as adjuvants to existing therapies or for the development of novel therapies for colorectal cancer.

The VICTOR study is a phase III, randomised double blind, placebo-controlled trial of the COX-2 selective inhibitor rofecoxib (VIOXX®) in colorectal cancer patients following potentially curative therapy. The VICTOR trial compares rofecoxib to placebo with the expectation that patients will experience increased overall survival and relapse free survival on taking rofecoxib for at least two years. The difference in the survival outcomes between two and five years of treatment is also under investigation. The study is an international trial, recruiting 7000 patients over 5 years. UK centres began recruitment in April of 2002. Centres in North America and Australasia will begin randomisation of patients in 2003. Study completion and final analysis is expected in 2012.

© 2003 Cancer Research UK

### P175

MOLECULAR ANALYSIS OF THE PDGFRα-RAS/MAPK PATHWAY IN CHILDHOOD MEDULLOBLASTOMA

Jacqueline A. Langdon*, Roberto Hernan, Jennifer Atkinson, Richard J. Gilbertson and Steven C. Clifford
Northern Institute for Cancer Research, University of Newcastle, Newcastle-upon-Tyne, U.K. & St Jude Children's Research Hospital, Memphis, TN, U.S.A.

Medulloblastoma (MB) is the most common malignant brain tumour of childhood. Current survival rates are 50-60% and clinical predictors of poor outcome include age (<3yrs) and presence of metastases. Molecular mechanisms underlying MB development may provide more informative insights into tumour biology and clinical behaviour; however, the molecular basis of this disease is poorly understood.

A recent microarray gene expression profile study of 23 MBs reported up-regulation of platelet-derived growth factor-α (PDGFR-α) and the downstream RAS/MAPK signal transduction pathway to be associated with advanced metastatic disease stage [MacDonald *et al.* (2001) Nat Genet. 29: 143-52]. Here, we studied genetic activation of this pathway in a panel of 28 primary MB and 9 MB cell lines by mutational analysis of *H*, *K* and *N-RAS* and *B-RAF.* No activating mutations were found in any of these genes. Western blot analysis identified moderate-high levels of PDGFR-α protein in 3/28 MB tumours; however, this did not correlate with metastatic disease or any other clinical feature. Our data suggest that activation of the PDGFR-α/RAS/MAPK pathway is an infrequent event in MB. We are currently analysing phospo-ERK levels and PDGFR-α mutational status in these same primary tumours to determine whether pathway activation occurs through alternative mechanisms.

### P176

THE ICR CLINICAL TRIALS & STATISTICS UNIT – AN OVERVIEW OF PRESENT WORK & FUTURE PLANS Lindsay Johnson*, Judith M Bliss, Emma Hall ICR-CTSU, Brookes Lawley Building, Institute of Cancer Research, Sutton SM2 5NG

During 2002, 2671 patients were randomised into Phase III multicentre trials conducted by the Clinical Trials & Statistics Unit at the Institute of Cancer Research (ICR-CTSU). Six multicentre trials are currently open to recruitment (TACT, HRT Trial, HERA, BC2001, Intercontinental, and the SLNB Melanoma Trial). 12434 patients are in active follow-up in multicentre trials now closed to recruitment (START, ABC, Topic I & II, Trafic, AHT, MSG-BAPS, Breast Dosimetry and Breast Fractionation. 7 multicentre trials are under development. Trials in 7 disease sites are conducted with breast as the specialist area of research activity.

The ICR-CSTU embraces the spirit of national collaboration by developing trials in conjunction with principal investigators and trials groups based throughout the UK. This is furthered by ensuring the involvement of the NCRI clinical studies groups.

A successful track record in building banks of biological material has been established, with large national collections of paraffin embedded material (ABC, TACT), and blood samples (START, TACT). Through this activity we have ongoing collaborations with established UK experts in translational research.

Our Phase III multicentre trials incorporate Quality of Life(QOL) studies, which form an integral component and are run from ICR-CTSU. QOL studies are developed in collaboration withnationally recognised QOL experts.

In keeping with our philosophy of national collaboration, we actively seek input from a broad base of UK clinicians and other UK trials groups in developing and running our trials. Effective mechanisms through which to acknowledge the contribution made by regional research

### P177

AN EVOLVING MODEL FOR FLEXIBLE TRIAL CO-ORDINATION – THE AZURE TRIAL

Liz Graham*, Vicky Napp, Stephanie Pollard
Northern & Yorkshire Clinical Trials & Research Unit,
University of Leeds, 17 Springfield Mount, Leeds, LS2 9NG

The AZURE Trial is a new international multi-centre randomised controlled phase III trial which aims to assess the benefits of adding adjuvant Zoledronic acid (bisphosphonate) to standard treatment for women with high risk localised breast cancer. 3300 patients are to be recruited over a maximum period of 3 years in order to answer this important question within a reasonable time frame.

The most effective way to achieve optimum recruitment rates was seen to be the implementation of the "hub and spoke" organisational model. This has been successful in a number of other large breast cancer trials. In the case of AZURE, the Northern & Yorkshire Clinical Trials & Research Unit are acting as co-ordinating centre ("hub"), working alongside other UK and international trials units ("spokes") who will take responsibility for local investigators and data management.

A similar model exists within the Yorkshire Breast Cancer Research Group, the "hub" in this case being the YBCRG, with "spokes" including research working groups, regional trusts, NCRN, NYCTRU, BIG and the EORTC. These links remain and will be integrated into the AZURE hub and spoke structure.

The NYCTRU intend to work flexibly with the aim of making recommendations as to the most effective organisational procedures. Anticipated benefits include strengthening of links within and between NHS trusts, cancer research networks/groups, and trials units across the country, with resulting improvement in communication, collaboration and recruitment.

### P178

ESTABLISHMENT OF A DNA RESOURCE THROUGH THE GELCAPS CONSORTIUM TO STUDY LOW PENETRANCE SUSCEPTIBILITY ALLELES FOR LUNG CANCER

A. Matakidou*, T. Eisen, M. O'Brien, R.S. Houlston and members of the GELCAPS Consortium. Sections of Medicine & Cancer Genetics, Institute of Cancer Research, Sutton, Surrey, SM2 5NG

There is increasing evidence for the existence of inherited susceptibility to lung cancer. It is likely that at least part of this is mediated by the action of low-penetrance alleles. Identification of genes in this class is contingent on association studies. To detect these alleles which confer small genotypic risks of around 1.5 requires large cohorts of cases and controls. Most studies reported to date have been under-powered and perhaps not surprisingly have produced discordant findings. To undertake a large-scale study of this nature, we have established the GELCAPS (**GE**netic **L**ung **CA**ncer **P**redisposition **S**tudy) Consortium to ascertain and collect blood samples and lifestyle information of 2,000 patients diagnosed with lung cancer in the UK. Coupled with this we are collecting a similar number of controls. To date we have collected 1,300 cases. Through this we shall have access to a systematic series of 2,000 unselected lung cancer cases and controls. Initially we are using this resource to evaluate the role of common variants within DNA repair enzymes and determinants of methylation status (MTHFR) using known and newly identified SNPs. Genotyping is being undertaken using TAQ man technology and preliminary results on the first 1,000 cases and controls will be analysed by August 2003 and presented at the meeting. Once technology permits, we shall use our resource for a genome-wide screen to identify novel low-penetrance lung cancer genes.

GELCAPS has been sponsored by Aventis and Dr Matakidou is supported by the Alan J Lerner Research Fund at the Royal Marsden Hospital.

**P179**

CANCER DIVISION, MRC CLINICAL TRIALSUNIT: SUMMARYOF TRIALS. Simon Weedon, Cancer Division, MRC Clinical Trials Unit, 222 Euston Road, London NW1 2DA (tel: 0207670 4700;fax: 020 7670 4818; email: contact@ctu.mrc.ac.uk;http://www.ctu.mrc.ac.uk).

The Cancer Division of the Medical Research Council Clinical Trials Unit designs, conducts and analyses many large randomised clinical trials covering most tumour sites.

Four trials will be presented in the Clinical Trials Showcase at this year's meeting: BO06, a randomised trial of chemotherapy with or without granulocyte colony-stimulating factor in operable osteosarcoma; ICON4, a randomised trial of paclitaxel in combination with platinum vs conventional platinum-based treatment in relapsed ovarian cancer; LU21, ifosfamide, carboplatin and etoposide with mid-cycle vincristine versus standard practice chemotherapy in patients with small cell lung cancer; and ST02 (MAGIC), a randomised controlled trial or pre- and post-operative chemotherapy in patients with operable gastric and lower oesophageal cancer.

We have recently opened three new trials: OE05, comparing chemotherapy regimens in patients with resectable adenocarcinoma of the oesophagus; BR12, temozolomide vs PCV chemotherapy in the treatment of recurrent malignant glioma; and MS01, active symptom control with or without chemotherapy in the treatment of patients with malignant pleural mesothelioma.

Two trials have recently closed: TIP, a study of paclitaxel, cisplatin and ifosfamide for metastatic germ cell tumour which has relapsed following BEP chemotherapy; and BS06, radical radiotherapy in the management of pT1G3 TCC in superficial bladder (BS06).

We have an extensive portfolio of open trials covering all major disease sites, these are listed on our poster.

**P180**

META-ANALYSIS GROUP, MRC CLINICAL TRIALS UNIT: SUMMARY OF RESEARCH.Clair vale, Meta-analysis Group, MRC Clinical Trials Unit, 222 Euston Road, London NW1 2DA (tel: 020 7670 4700; fax: 020 7670 4816; email: metaanalysis@ctu.mrc.ac.uk; http://www.ctu.mrc.ac.uk).

The Meta-analysis Group of the Medical Research Council Clinical Trials Unit designs and conducts systematic reviews and individual patient data (IPD) meta-analyses in a variety of diseases, notably cancer. We work closely with the Cochrane Collaboration, both in the conduct of systematic reviews and meta-analyses and also in methodological research. The Group also coordinates a national register of randomised cancer trials, (formerly the UKCCCR Register) and is involved in a number of initiatives in trial registration.

The results of one IPD meta-analysis will be presented at this year's meeting, in the Current Controversies in Gynaecological Cancer Symposium: "Is neoadjuvant chemotherapy of benefit in the treatment of locally advanced cervical cancer?"

Highlights of other projects will be detailed in this poster, including: results of a recently completed IPD meta-analysis of neoadjuvant chemotherapy in invasive bladder cancer, progress reports on ongoing and forthcoming IPD meta-analyses and summaries of our portfolio of methodological research.

The poster will also outline developments in trial registration, drawing attention to recent improvements to the cancer trials register and its website, featuring the new-look site with enhanced facilities for trial registration.

**P181**

DOCUMENTATION ASSOCIATED WITH USE OF IV PALLIATIVE CHEMOTHERAPY (PC) - AN EXAMPLE OF THE USE OF THE AUDIT PROCESS TO CHANGE CLINICIAN BEHAVIOUR. *Jean.Abraham, Astrid.Mayer, Gemma.Whiting, Helena.Earl, Margaret.Moody. Oncology Dept Addenbrookes NHS Trust, Cambridge CB2 2QQ.

**Aims:** An initial audit was performed in 1999 to ascertain if IV PC was given according to departmental guidelines and to assess adequacy of associated documentation. The results required in documentation. Use of toxicity flow sheets was recommended. The current audit was used to assess whether the changes implemented had achieved improved documentation.
**Method:** 75 randomly selected notes of non-trial patients (upto 10 per consultant) who received IV PC between 05/01 to 05/02 were audited. Documentation data, collected by means of a pro forma, included: Performance Status (PS) (each cycle); treatment toxicity (TT) (each cycle); symptoms documented (before present cycle); reasons for giving chemotherapy; evaluable disease; number of cycles planned; interim response assessment (planned or completed); location of primary; current regimen.
**Results:** A comparison of current (CA) and previous (PA) audit results showed documentation of; PS 77% (CA) 23% (PA); TT 96% (CA) 62% (PA); Symptoms 98% (CA) 83% (PA);reason for giving chemotherapy 92% (CA) 89% (PA); evaluable disease 95% (CA) 96% (PA).
**Conclusion:** There was a marked improvement of documentation overall. The implementation of toxicity flowcharts as recommended by the 1999 initial audit significantly improved the level of adequacy of documentation where used.

**P182**

EXPECTATIONS COMPARED TO ACTUAL QUALITY OF LIFE IN PATIENTS WITH HEAD AND NECK CANCER Roger. Wheelwright*, Martin A. Birchall University of Bristol, Medical School Unit, Southmead Hospital, Bristol BS10 5NB, England, UK

**Background** Past outcome measures have mainly to date been based on 5 year survival now increasing attention is being paid to quality of life (QoL) as an outcome tool. **Overview** As part of a Critical Path Analysis study one objective was to address the question: Does patient QoL at four months and one-year match their expectations at pre-treatment stage?
**Subjects** Patients for this study were those presenting for the first time with head and neck cancer to the centres during a 12-month study period.
Quality of Life tools: EORTC with Head and Neck Module, Hospital Anxiety and Depression Scale (HADS), two expectations and actuality questions added, questionnaire given at 1st OPA, 4 months and 12 months
Expectations and Actuality: 1st Questionnaire: What do you expect your overall physical condition will be in 12 months, after your treatment? and What effect do you think the treatment will have on your overall Quality of Life in 12 months time?
Expectations and Actuality: 4 months/1yr Questionnaire: 4/12 months ago, before your treatment, what did you expect your physical condition to be now? and 4/12 months ago, what effect did you think the treatment will have on your overall Quality of Life?
**Results** Analysis of the patient scores demonstrated low scores for anxiety and depression, with no indication of clinical depression amongst the subject group.
Limitations of findings: Sample size small n=21, Results from one centre
**Conclusions** A high level of anxiety and depression with low expectations was anticipated with this group of patients, the results indicate otherwise. Although interesting, definitive conclusion cannot be made for the quality of life findings, a further study of quality of life needs to be done with a larger cohort of patients.

© 2003 Cancer Research UK

**P183**

A STUDY TO EVALUATE THE USE OF CA125 IN OVARIAN CANCER (EOC) FOLLOW-UP (FU). A CHANGE IN PRACTICE LED BY PATIENT PREFERENCE Jennie Pratt, Cheryl Palmer, Bristi Basu, Jean Abraham, Swethajit Biswas, James Brenton, Helena Earl*. Dept. of Oncology, Addenbrookes Hospital Cambridge CB2 2QQ

**Introduction:** Pre-study, women on FU for EOC had blood taken for Ca125 at their clinic visit (Ca125-FU). Symptomatic patients were informed of the result by phone, asymptomatic patients with elevated or normal Ca125, might be informed at their next FU. **Patients and Methods:** Patient questionnaires (Q) were developed including questions exploring their understanding of Ca125, satisfaction with current service, and whether they would prefer to have an up to date Ca125 measurement available in clinic. A change in practice was then implemented, and a repeat Q was sent to survivors two years later to assess their satisfaction with this. **Results:** Qs were sent to 100 women with EOC attending the centre for follow-up and 88% replied. Only 50% of women were satisfied with Ca125-FU. 81% documented that they would prefer the current result to be available in the clinic. A change in practice was instituted. Local GPs were contacted and 100% agreed to carry out Ca125, 2 weeks prior to FU, using a pre-prepared pack provided by the centre. Women were informed of their result at their next clinic visit. Two years later, a repeat Q was sent to the 37 survivors. 92% reported improved quality of follow-up by the change in practice. 92% reported their results were available in clinic (88% 'always', and 4% 'usually'). The repeat Q also reported that 88% of women had an understanding of Ca125-FU compared with 81% from the initial study. **Conclusions:** There was a marked improvement in patients' perceptions of the quality of Ca125-FU as a result of a successful change in practice led by patients' preference.

**P184**

CANCER CHEMOTHERAPY SERVICES IN GRAMPIAN, ORKNEY AND SHETLAND: GOOD, BAD AND UGLY.
Marianne Nicolson*, Susan Healy, Lynn Adams, Tanya Learmonth and Elaine Neil, Aberdeen Royal Infirmary, AB25 2ZN.

Introduction: Throughout the NHS there is a drive to improve standards for treatment delivery through development of evidence-based protocols. The safe prescription and administration of chemotherapy is one area where there is a need for specialisation and close collaboration with all concerned practitioners. Primary and secondary care physicians, community and hospital nurses, pharmacists and other staff must all be fully aware of the safety issues concerned and their role in maintaining good standards of care. On behalf of the Grampian Medicines Committee Oncology Subgroup, an audit of local cancer chemotherapy services was carried out between 25$^{th}$ March and 18$^{th}$ May 2002.

Method: The audit tool was the document produced by the Scottish Cancer Care Pharmacy Group (SCCPG). This assesses standards set out by the document 'Safe use of cytotoxics in the clinical environment' recommended by the Scottish Executive (HDL 2001.13). There was also a spot review of casenotes in the areas visited. Two members of the audit team visited each of the twelve areas where chemotherapy is administered. Tight working definitions of the specific standards set by the SCCPG document were agreed. Factors addressed, relating to cytotoxic chemotherapy, were prescribing (prescriptions and protocols), preparation and supply, administration, disposal and minimising exposure. Each subsection of these headings was allocated a Grade of 1 where there was full compliance with the standard, 2 for minor non-compliance, 3 for non-compliance and 4 if non-compliance was major or critical.

Results: Overall Grade 1 was achieved in less than 50% for all but the disposal standard. Details and progress will be presented.

**P185**

INCREASE FROM LOW TO MODERATE DOSE-RATE IN GYNAECOLOGICAL BRACHYTHERAPY AND DOSE CORRECTION- A RETROSPECTIVE ANALYSIS OF PATIENTS TREATED AT WESTON PARK HOSPITAL.
Jayaram Mohanamurali*, David J. Radstone, Simon D. Pledge, Weston Park Hospital, Witham Road, Sheffield, S10 2SJ.

Although there is a general consensus amongst radiotherapists that a dose reduction is needed to compensate for an increased dose-rate in brachytherapy, the magnitude of the dose reduction required still remains a subject of considerable debate.

Clinical trials which have compared various schedules suggest an optimum dose reduction to be about 10-12%, for a change from low to moderate dose-rate.

At Weston Park Hospital, conservatively, the total dose to point-A was reduced by 20% in patients treated with the selectron remote afterloading technique, compared to those treated pre-selectron, to compensate for the increase in dose-rate in 1994.

245 case notes of patients with carcinoma of the cervix, registered with Weston Park Hospital in 1992, 1993, 1995 and 1996 were reviewed. A total of 35 patients treated with two insertions of brachytherapy followed by external beam boost to the parametria were identified and included in the analysis. 20 patients were treated pre-selectron and 15 patients were treated using selectron with the above regime.

The mean point-A dose-rate in the pre-selectron group was 87.55 cGy/hr and 135.47 cGy/hr in the selectron group. The ratio of the change in dose-rate was 1.55. There was no statistically significant difference between the two groups of patients, with regards to local recurrence rates or morbidity.

Despite only a modest increase in dose-rate at point-A, the reduction in the total dose prescribed at point-A by 20% to compensate for the increase in dose-rate does not appear to have an adverse impact on local control in this small group of patients.

**P186**

CHARACTERISTICS OF ENDOMETRIOID AND CLEAR CELL EPITHELIAL OVARIAN CANCER: AN ANALYSIS OF PROSPECTIVELY COLLECTED DATA 1984-2001Dawn J.Storey*, Moira Stewart, Tyzvia Rye, Awatif Al-Nafussi, Alistair R Williams, John F.Smyth, Hani Gabra. Cancer Research UK, University of Edinburgh Cancer Research Centre, Crewe Road South, Edinburgh, UK.

**Background:**There is little published data on endometrioid ovarian tumour response rates to platinumbased chemotherapy (PBC).Previous published work suggested that the clear cell (CC) variant of ovarian carcinoma only had an 11% response rate as compared to serous tumours (72.5%). Survival was also poorer. We have evaluated the characteristics, PBC response rates and outcomes of our Endometrioid(E) and CC patients.

**Methods:** Between 1984-2001, after central pathology review, data was prospectively collected for 267 patients with pure E, and 88 patients withpure CC ovarian cancers.

**Results:** Age, stage distribution, surgical debulking status, performance status and proportion receiving PBC were the same for both tumour subtypes. E tumours had significantly more concurrent uterine malignanciesthan CC (p=0.024). Of those who received chemotherapy 165/194 E patientsreceived PBC. 103/194 had radiological or Rustin Ca125 evaluable disease after surgery. PBC response rates were significantly higher for E tumours (63%) than for CC (36%,p=0.001). Overall survival and progression free survival was significantly better for E tumours with stage III disease compared with CC (p= 0.001and 0.004 respectively). Debulking to <2cmsignificantly improved survival for both CC and E (p<0.0001).

**Conclusion**: Response rates to PBC and survival for stage III are better inE tumours than CC. This analysis may have implications for future trial designs.

© 2003 Cancer Research UK

**P187**

A SINGLE CENTRE AUDIT OF BLADDER CANCER. Jeetesh M Bhardwa*, Bharat K Sangtani, F I Chinewundoh, A M I Paris, C G Fowler, Vinod H Nargund

Aim – To quantify the in patient work generated due to bladder cancer (TCC) and the need to develop new markers to monitor it.

Method – Retrospective audit of in patient activity due to a diagnosis of TCC for the years 1998, 1999,2000 at the Urology department at St. Bartholomews Hospital, London.

Findings - There were 433 patients who had a total of 1188 admissions/ episodes (An average of 2.7 episodes per patient).

Patient demographics - Average age = 69.9 years (Median = 71 years). Range = 31 years to 91 years. Male = 327 (75%) Females = 106 (25%)

Procedures carried out in three years were as follows:

TURBT = 548, Cystoscopy (under GA) = 168, Cystodiathermy (under GA) = 168; Cystectomy = 14.

That is an average of 295 endoscopic procedures per year.

A total of 264 instillations of intravesical Chemotherapy in three years were performed. This was received by 52 patients in all with an average of 5.1 instillations per patient.

Conclusion - A lot of resources are spent in treating and monitoring bladder cancer patients. If all the flexible cystoscopies and Out Patients appointments are taken into consideration then the impact on the resources is even greater. This data justifies further research into markers of bladder cancer. In particular superficial bladder cancer that is at high risk of progressing to muscle invasive disease needs to be differentiated from that which is at low risk. These patients can then be followed up more efficiently and effectively.

**P188**

EARLY (60-DAY) MORTALITY RATES, IN GI CANCER PATIENTS TREATED WITHIN RANDOMISED TRIALS, AT THE ROYAL MARSDEN HOSPITAL.

Ourania Katopodis*, Paul J Ross, Andy R Norman, David Cunningham. Royal Marsden Hospital, Sutton, Surrey, SM2 5PT

Aim: To document the 60 day all cause mortality rate, during chemotherapy, for patients with oesophagogastric (O-G), pancreatic (panc.) and colorectal (CRC) cancer.

Procedure: We conducted an analysis of 1720 patients with a minimum follow up of 60 days treated within randomised trials. Kaplan Meier survival curves were used to calculate the all cause mortality and 95% confidence intervals (CI). Causes of death were classified as treatment related (toxicities), disease related or vascular syndrome (MI, CVA or PE). Patients with O-G cancer were divided into those treated with platinum containing combinations and those unable to tolerate it. These patients received infused 5FU with or without mitomycin C.

Results: (Table)

Significance: This study provides a bench mark for assessing the safety of regimens used in these disease settings.

Conclusions: Site of primary determines 60 day mortality. The rate of treatment related and vascular syndrome induced deaths is low (<1.8%). The majority of deaths within 60 days, in patients with advanced disease, were disease related. Only 1 patient (0.2%) died in the first 60 days (cause of death: MI) of adjuvant chemotherapy for CRC.

| Group | N | 60 day mortality | CI | Treatm. related deaths | Disease related deaths | Vascular syndrome deaths |
|---|---|---|---|---|---|---|
| Advanced CRC | 609 | 3.4 % | 2.3 - 5.2 | 0.1 % | 3.3 % | 0 % |
| Adjuvant CRC | 577 | 0.2 % | 0.0 - 1.2 | 0 % | 0 % | 0.2 % |
| O-G Platinum | 254 | 6.3 % | 3.9 - 10.1 | 0.4 % | 4.7 % | 1.2 % |
| O-G Non platinum | 109 | 8.3 % | 4.4 - 15.3 | 0 % | 7.4 % | 0.9 % |

 © 2003 Cancer Research UK

